# Stage-dependent role of NEK7 in the inactive-to-active conformational transition of NLRP3 monomer

**DOI:** 10.1371/journal.pcbi.1014405

**Published:** 2026-06-12

**Authors:** Jin Peng, Wenjian Li, Hao Wang, Xiaohui Chen, Manjie Zhang, Bin Sun

**Affiliations:** 1 Research Center for Pharmacoinformatics, College of Pharmacy, Harbin Medical University, Harbin, China; 2 Department of Pharmaceutics, College of Pharmacy, Harbin Medical University, Harbin, China; Max Planck Institute of Molecular Plant Physiology: Max-Planck-Institut fur molekulare Pflanzenphysiologie, GERMANY

## Abstract

The NLRP3 inflammasome is a multiprotein complex that primes cytokine production in the innate immune system. The inflammasome activation involves the cage-to-disk transition of NLRP3 oligomers, facilitated by the co-factor NEK7 protein. While NEK7’s role in promoting cage disassembly has been reported, its involvement in the large conformational changes of the NLRP3 monomer during activation remains elusive. Here, by using multi-scale simulations, we uncovered a stage-dependent role of NEK7 in the inactive-to-active transition. In the early stage, NEK7 reshapes the dynamics of the highly unstable inactive NLRP3 monomer to resemble active state, priming the conformational transition. In the middle stage, NEK7 impedes progression by populating an intermediate state farther from the active conformation than the NEK7-free counterpart, and structures in this state exhibit reduced allosteric potential toward activation. In the late stage, NEK7 has negligible impact, as the active conformation remains inherently isolated by a high energy barrier regardless of NEK7 presence. This highlights the critical role of oligomeric assembly in enabling monomeric NLRP3 to complete its conformational transition, in agreement with experiment observations. Our work suggests a multilayered activation mechanism where oligomer-level assembly and monomeric conformational changes are coupled, providing new mechanistic insights into this physiologically essential macromolecular process.

## Author summary

The NLRP3 inflammasome is an important sensor of the innate immune system, whose dysregulation underlies various inflammatory diseases, making it a major therapeutic target. While the protein NEK7 is implicated in NLRP3 inflammasome activation, its precise molecular role has remained unclear. This study used multi-scale simulations to show that NEK7 plays a stage-dependent role in driving the large conformation transition of the NLRP3 monomer from its inactive to active form, a process that has been difficult to capture experimentally. We showed that NEK7 first primes the inactive protein to begin its transition, then transiently hinders it at an intermediate stage, and finally becomes irrelevant as a high energy barrier prevents the monomer from completing activation on its own. This work deepens our understanding of inflammasome activation by demonstrating that the final, critical step of conformational change of the NLRP3 monomer is unlikely to occur without the assembly of multiple NLRP3 proteins into a larger oligomeric complex. This study suggests that targeting the early or intermediate steps of NLRP3 activation may have therapeutic potential.

## 1. Introduction

NLRP3 is a vital mediator of the innate immune system, responding to exogenous microbial invasion and endogenous damage signals [1,2]. As a pattern recognition receptor (PRR), NLRP3 orchestrates the formation of a multiprotein complex called the inflammasome to generate cytokines upon sensing danger signals. The activated NLRP3 inflammasome recruits the apoptosisassociated speck-like protein containing a caspase-recruitment domain (ASC), activating caspase-1, which cleaves cytokine precursors to generate mature cytokines such as interleukin (IL)-1β and IL-18, ultimately triggering cell damage and death [2]. NLRP3 inflammasome can be activated by diverse stimuli such as nigericin, uric acid crystals, amyloid-β fibrils, and extracellular ATP [3]. Since abnormal NLRP3 activation (e.g., due to mutations) is closely linked to autoimmune disorders, NLRP3 is a validated therapeutic target, and inhibitors are under active development as anti-inflammatory drugs [4–6].

**Fig 1:**
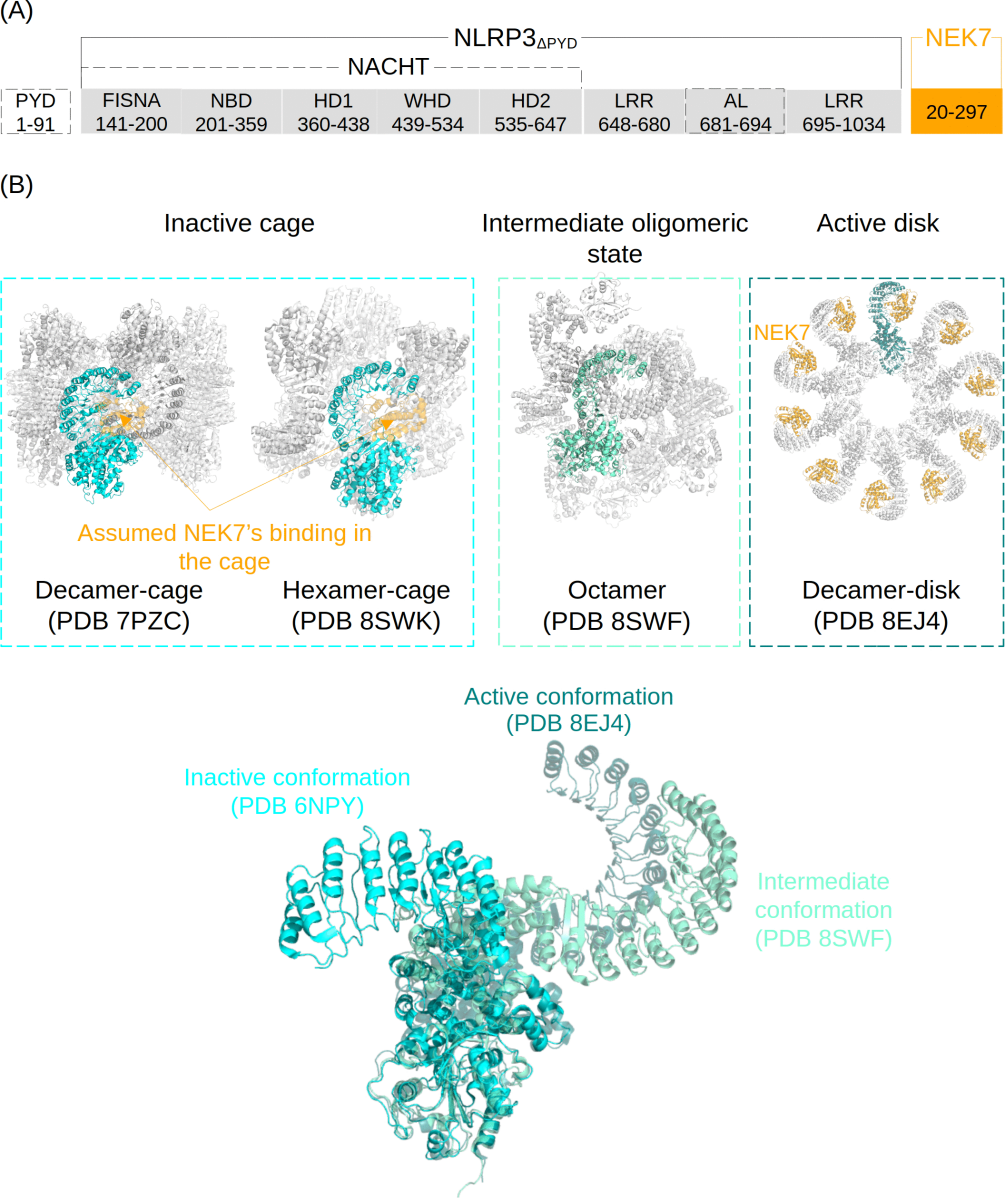
Structural background. **(A)** Domain organization of human NLRP3 protein. This study utilizes the NLRP3_ΔPYD_ construct and NEK7_20−297_ construct. **(B)** NLRP3 inflammasome activation involves oligomer-level assembly coupled with monomer-level conformational changes. While NEK7’s oligomeric role (e.g., promoting dissociation of the inactive cage) has been suggested [1–3], its impact on monomeric NLRP3 conformational transitions remains unknown. In this study, by using multiscale computational modeling techniques, we aim to explore the impact of NEK7 on the conformational transition of monomeric NLRP3.

Elucidating the structural basis of NLRP3 inflammasome activation is critical for therapeutic development. The human NLRP3 monomer is a 1036-residue multidomain protein consisting of an N-terminal pyrin domain (PYD), a nucleotide-binding and oligomerization domain (NACHT), and a C-terminal LRR domain [7] (Fig 1A). Recent Cryo-EM studies have revealed that NLRP3 proteins exist in physiologically important oligomeric forms in both inactive and activated states (Fig 1B). The inactive NLRP3 forms a double-ring cage, an oligomeric structure necessary for its transport to the centrosome for subsequent activation [8]. Similarly, the activated inflammasome adopts a disk-shaped oligomer [3], which facilitates PYD filament formation with ASC proteins and subsequently primes cytokine production. Thus, at the oligomer level, NLRP3 inflammasome activation is marked by the transition from the inactive cage to the active disk, and NLRP3 mutations that either destabilize the cage or stabilize the disk can cause pathogenic NLRP3 hyperactivation [9].

In the cellular environment, the hydrolysis of ATP can transform the inactive “birdcage-like” decamer into the active octamer [10]. In addition, a key co-factor, the NEK7 protein, plays a vital role in the oligomeric cage-to-disk transition of the NLRP3 inflammasome. NEK7 is a member of the NIMA-related kinase (NEK) family that has been shown essential for NLRP3 inflammasome activation [1,11,12]. Cryo-EM studies suggest that NEK7 binding to the inactive NLRP3 monomer could induce steric clashes with neighboring NLRP3 monomers within the cage [10] (Fig 1B), promoting inactive cage disassembly. This NEK7-mediated cage disruption releases NLRP3 monomers for subsequent disk-shaped oligomer assembly, underscoring NEK7’s critical role at the oligomer level.

Recent work has further clarified both the existence of NLRP3 monomers and the importance of the NEK7–NLRP3 interaction. Boršić *et al* [13] showed that the membrane can promote NLRP3 clustering to allow inflammasome activation even in the absence of canonical activators; their model presumes that inactive NLRP3 can exist either in cage-like structures or as monomers. Thus, while the end point of activation is a disk-like oligomer, the starting state of the inactive form can vary from monomers to dimers to larger assemblies. Regarding NEK7 function, three recent studies provide compelling evidence: Zhang *et al* [12] and Jin *et al* [14] demonstrated that disrupting the NEK7–NLRP3 interaction inhibits inflammasome activation, whereas Xu *et al* [15] showed that phosphorylated NEK7 enhances NEK7–NLRP3 binding and accentuates activation.

Despite these insights into NEK7’s oligomer-level function, its impact on the monomeric NLRP3 conformation remains unclear. NLRP3 inflammasome activation involves not only the cage-to-disk transition but also large conformational changes in the monomer. Since both inactive and active NLRP3 monomers can bind NEK7 [1,3], whether and how NEK7 influences the monomer’s conformational transition is unknown. Importantly, although NLRP3 operates primarily in oligomeric forms under physiological conditions, from a biophysical standpoint these oligomers are composed of individual NLRP3 monomers. The monomer serves as the basic building block of the oligomeric inflammasome; therefore, understanding the internal dynamics of the NLRP3 monomer is directly relevant to the structural activation mechanism of the higher-order complex.

Monomeric NLRP3 adopts different conformations in the inactive cage versus the activated disk oligomer, characterized by an ∼90° rotation of the WHD-HD2-LRR domain relative to the FISNA-NBD-HD1 domain [1] (Fig 1B). Although activated NLRP3 binds NEK7 in the disk-shaped oligomer, it is the N-terminal NACHT domain of NLRP3 that nucleates the disk while the LRR-NEK7 interaction regions are extending toward the disk periphery (Fig 1B). This implies that NEK7 may have minimal influence on the activated NLRP3 conformation. However, a recent study resolved an intermediate oligomeric state of the NLRP3 inflammasome (PDB: 8SWK [10]), in which NLRP3 adopts a novel conformation distinct from the inactive and activated states. This intermediate hexameric state (Fig 1B) implies that NLRP3’s monomeric conformational changes and oligomeric cage-to-disk transition are coupled. Given that monomeric NLRP3 can adopt multiple conformations, understanding NEK7’s role in NLRP3’s conformational transition, and its implications for inflammasome oligomerization, is crucial, as NLRP3’s structural properties directly influence immune response outcomes [16].

Several studies have explored the conformational and dynamic profiles of monomeric NLRP3. For instance, Casali *et al* [17] investigated how ADP and inhibitor binding affect NLRP3 dynamics via molecular dynamics (MD) simulations, showing that these ligands induce different dynamic behaviors. El-Sayed *et al* [18] used unbiased and accelerated MD (AMD) simulations to examine NEK7’s effect on inactive NLRP3 monomer, demonstrating that inactive NLRP3 exhibits high plasticity in the presence of NEK7, and that NEK7 removal leads to monomer compaction. A recent study by Xu *et al* [19] employed MD simulations to demonstrate that NLRP3’s conformational transition is intertwined with oligomer assembly, confirming that intermonomer interactions convert an energetically uphill transition into a downhill process, highlighting the importance of higher-order assembly in activation.

In this study, we employed multi-scale molecular dynamics simulations to investigate NEK7’s influence on the conformational transition of monomeric NLRP3 from its inactive to active state. By integrating conventional MD and biased sampling techniques, we constructed the transition landscape of NLRP3 with and without NEK7, uncovering its stage-dependent regulatory roles. Specifically, at early stage, NEK7 reshapes the dynamics of inactive NLRP3 to resemble that of the active state, promoting transition initiation. At middle stage, NEK7 stabilizes intermediate states farther from the active conformation, exhibiting a slightly inhibitory effect. And at late stage, NEK7’s influence diminishes as intrinsic energy barriers separate the active conformation regardless of NEK7 presence. These barriers highlight the essential role of oligomeric assembly in completing the transition. Our findings support a multi-layered activation mechanism where monomeric conformational plasticity and oligomeric restructuring are coupled, advancing mechanistic understanding of NLRP3 inflammasome activation.

## 2. Results

### 2.1. Inactive and active NLRP3 monomers have different binding modes toward NEK7

**Fig 2:**
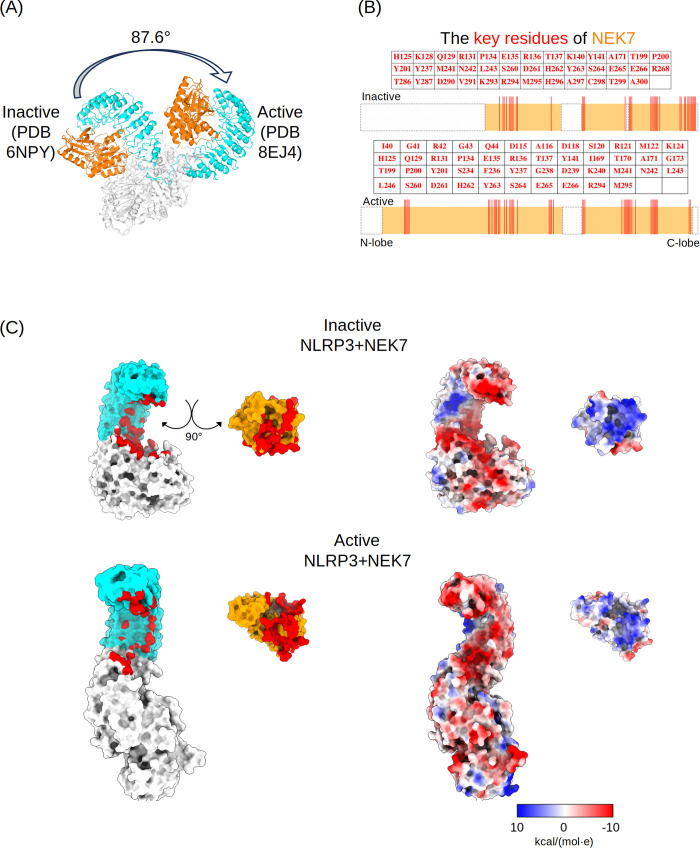
NEK7 binding modes with inactive and active NLRP3 monomers. **(A)** Structural overlay of inactive (PDB 6NPY) and active NLRP3 (PDB 8EJ4) monomer conformations aligned by their NACHT domains, highlighting large-scale conformational changes during activation. NLRP3 domains are colored as follows: NACHT (gray), LRR (cyan). NEK7 is shown in orange. **(B)** Key NEK7 residues that are within 5 Åof NLRP3. Residue positions are marked by red lines on the NEK7 sequence, with unresolved regions indicated by dashed boxes. **(C)** Open-book view of the NLRP3-NEK7 interface. Contacting residues (within 5 Å) are highlighted in red. Electrostatic potential surfaces (right) illustrate charge complementarity at the binding interface. Specific hydrogen bonds between NEK7-NLRP3 are listed in S1 Fig.

We first analyzed the binding modes of NLRP3-NEK7 in the inactive and active states to determine whether their interaction changes during NLRP3’s conformational transition. We characterized these modes by evaluating binding surface areas and identifying key intermolecular interactions. As shown in Fig 2A, Cryo-EM studies confirm that both inactive and active NLRP3 monomers bind NEK7. We further compared the binding modes between NEK7 and NLRP3 in these two states (Fig 2B). In the inactive state, NEK7 interacts with NLRP3 exclusively via its C-lobe, while in the active state, both the N- and C-lobes contribute to binding (Fig 2B). The interacting NLRP3 domains also differ: the inactive state involves both the NACHT and LRR domains, whereas the active state primarily engages the LRR domain. Quantitatively, the NLRP3-NEK7 interface in the inactive state (1665 Å^2^) is significantly larger than in the active state (1064 Å^2^), underscoring these structural differences. Despite these variations, binding surfaces in these two binding modes are electrostatically complementary. As shown in Fig 2C, NLRP3 and NEK7 exhibit complementary charged interfaces-NLRP3 is predominantly negatively charged, while NEK7 is positively charged-consistent with their reported isoelectric points (pI 6.2 vs. 8.5, respectively; [1]). This charge complementarity suggests physiological relevance for both binding modes. The different NLRP3-NEK7 binding modes in the inactive and active states demonstrate conformational transition-dependent interactions, implying NEK7’s multifaceted role in regulating monomeric NLRP3’s conformational transition.

### 2.2. The inactive NLRP3 monomer is structurally unstable but partially stabilized by NEK7 binding, whereas the activated monomer is intrinsically stable and unaffected by NEK7

**Fig 3:**
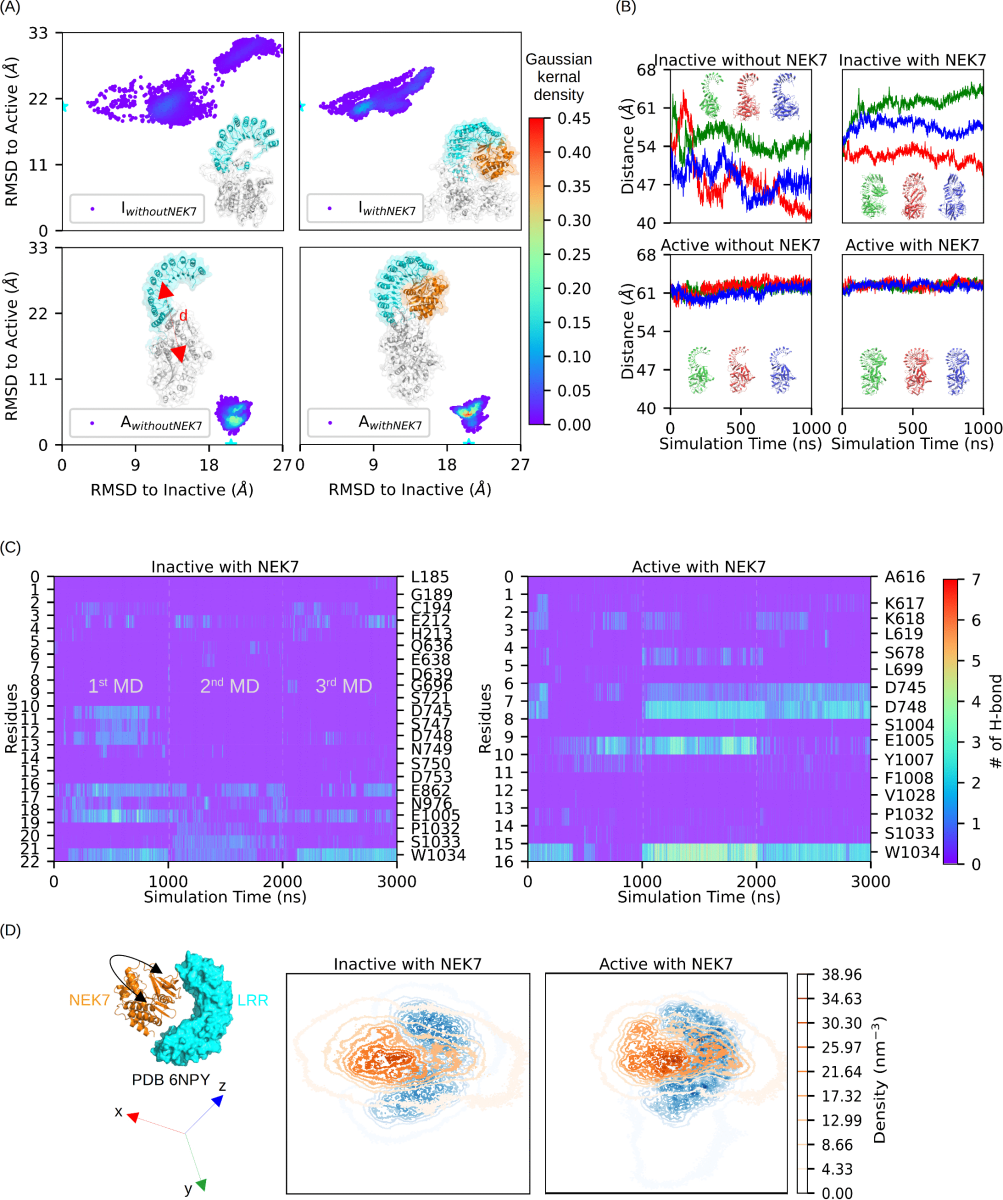
Effects of NEK7 on the end-state conformational stability of NLRP3. **(A)** Projection of three 1-μs conventional MD trajectories onto a 2D subspace defined by RMSD values relative to the inactive (x-axis) and active (y-axis) states of NLRP3. Visual inspection of the trajectories informed the definition of a distance metric, *d*, representing the separation between the centers of mass (COMs) of the LRR and NACHT domains of NLRP3. This metric characterizes key structural changes during simulations. **(B)** Time-dependent evolution of *d* across the three 1-μs MD simulations. **(C)** Time-dependent intermolecular hydrogen bond counts between NEK7 and NLRP3. For each NLRP3 residue engaged in hydrogen bonding with NEK7, the number of bonds formed over time is plotted. **(D)** Atom density projection onto the xy-plane calculated from MD trajectories using the *gmx densmap* tool. Trajectories were aligned to the LRR domain of PDB 6NPY with fixed orientation prior to density calculations.

To explore the effects of NEK7 on the stability of NLRP3 monomers, we performed 3×1 μs atomistic MD simulations on both the inactive and active NLRP3, considering each with or without NEK7 bound. We first projected the MD samplings onto the 2D conformational space using their RMSD values relative to the initial inactive and active state conformations, respectively. As shown in Fig 3A, the distributions of the two inactive systems, regardless of NEK7 presence, span a broad area, in contrast to the much more concentrated distributions of the two active systems. This suggests that monomeric NLRP3 is unstable in the inactive state conformation. Specifically, the RMSD (to inactive) values of the two inactive states span a wide range (5–15 Å), reflecting large structural deviation from the initial inactive conformation. Notably, these structural changes do not approach the active state, as the RMSD (to active) values remain consistently >20 Å, indicating that monomeric NLRP3 in the inactive conformation, despite its high flexibility, does not spontaneously transition to the active state. Meanwhile, the two active states exhibit sampling closely clustered around the initial structure, with RMSD (to active) ≤ 6 Å, demonstrating the stability of the active conformation with or without NEK7.

To further characterize the structural lability of inactive-state NLRP3, we defined a distance metric, *d*, as the center-of-mass (COM) distance between the LRR and NACHT domains to capture major structural variations. As shown in Fig 3B, inactive NLRP3 without NEK7 exhibited the largest fluctuations of *d*, along with a clear declining trend across all three independent MD runs, reflecting structural collapse due to instability. In the presence of NEK7, fluctuations were reduced, but the average *d* values varied across the three 1 μs runs, suggesting that NEK7 modestly stabilizes inactive NLRP3 while preserving its inherent lability. Radius-of-gyration analysis further confirmed the compaction of inactive NLRP3 monomers and the stabilizing effect of NEK7 (S2 Fig). In contrast, activated NLRP3 monomers maintained stable distance curves (∼61 Å) regardless of NEK7 presence, underscoring the high stability of the active state. Our observations align with EI-Sayed et al.’s MD study, which reported high plasticity in the inactive monomeric NLRP3 and compaction upon NEK7 removal [18]. Thus, the inactive state of NLRP3 is highly unstable without NEK7, while NEK7 binding provides partial stabilization. Conversely, the active state remains stable irrespective of NEK7.

Fig 2 suggests that NEK7’s binding mode depends on NLRP3’s conformation, as the interfaces differ between the two end states. We further analyzed NLRP3-NEK7 binding patterns during the MD simulations to assess interaction stability. Fig 3C shows time-dependent intermolecular hydrogen bonds during simulations. In both states, NLRP3 and NEK7 lack a fixed interface, instead employing diverse residues for hydrogen bonding. Compared to active NLRP3, NEK7 binding to the inactive state is dynamic, with shorter hydrogen bond lifetimes and frequent residue switching. Nevertheless, despite this plasticity, the LRR domain of NLRP3 and NEK7 maintain their bound configuration as seen in Cryo-EM structures (Fig 3D, 2D atom density). However, in inactive NLRP3, residues in the LRR, NEK7, and their interface are less densely packed relative to the active state (Fig 3D), consistent with a more dynamic NEK7-NLRP3 binding pattern in the inactive state. **Therefore, the inactive NLRP3 monomer is highly flexible, with NEK7 binding providing stabilization through a dynamic interaction. In contrast, the activated NLRP3 is intrinsically stable, and NEK7 binding has minimal impact on its conformation.**

### 2.3. NEK7 shapes the dynamics of inactive NLRP3 conformation to be active state-like

**Fig 4:**
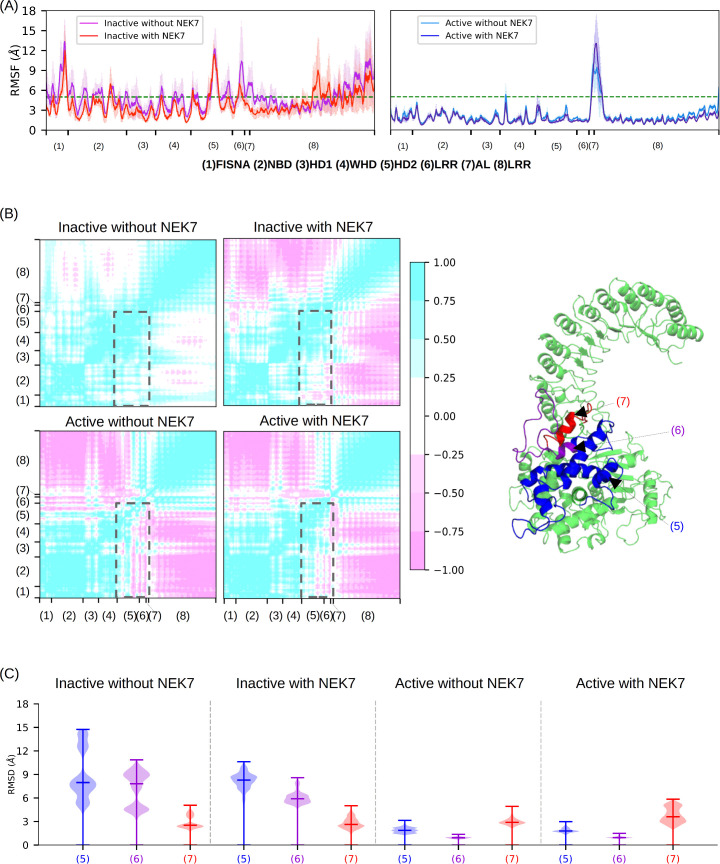
Effects of NEK7 on the dynamics of two end states of NLRP3. **(A)** RMSF of NLRP3 calculated from three independent conventional MD simulations. Shaded regions represent the standard deviation across simulations. **(B)** Dynamic cross-correlation matrix (DCCM) of NLRP3. The dashed box highlights the hinge region in PDB 6NPY, where the acidic loop (AL) is modeled by AlphaFold2 and is colored red which comprises residues 681-694. The blue, purple and red regions belong to the HD2 motif that precedes the AL. In these regions where positive and anti-correlated motions coexist. **(C)** Impact of NEK7 on conformational stability of the AL and HD2 regions, quantified by RMSD values relative to the starting MD simulation structures.

We now investigate how NEK7 influences the dynamics of the NLRP3 monomer in its two end states. Fig 4A shows the root-mean-square fluctuation (RMSF) of NLRP3 residues across the four systems. Consistent with our conformational analysis, isolated inactive NLRP3 is highly dynamic, with multiple motifs exhibiting large RMSF values (≥5 Å), whereas the active NLRP3 monomer is less flexible, with most residues have RMSFs around 2.5 Å. Interestingly, NEK7 binding modulates the dynamics of inactive NLRP3, suppressing fluctuations in the NACHT domain while amplifying dynamics in the C-terminal half of the LRR domain. In contrast, the dynamics of active NLRP3 monomer remain nearly unaffected by NEK7. These findings suggest that the inactive state is dynamic but can be partially stabilized by NEK7, while the activated state represents an optimized, stable conformation resistant to NEK7 perturbation.

Given NEK7’s effect on inactive NLRP3 dynamics, we explore how this modulation could influence allosteric signaling within the monomer. We calculated the dynamical cross-correlation matrix (DCCM) for all four systems (Fig 4B). The two active systems exhibit almost identical DCCM patterns, characterized by strong anti-correlations between the LRR and NACHT domains and positive correlations within each domain. These patterns reflect the allosteric signaling features of the activated NLRP3 monomer. In contrast, NEK7 binding alters the DCCM of inactive NLRP3. Isolated inactive NLRP3 lacks the LRR-NACHT anti-correlations observed in the active state, indicating that its dynamics, though being flexible, are not functionally productive toward activation. Interestingly, NEK7 binding reshapes inactive NLRP3’s dynamics to resemble those of the active state, inducing strong LRR-NACHT anti-correlations. This suggests that NEK7 primes inactive NLRP3 for transition to an activation-competent conformation.

A zoomed-in view of the DCCM data in Fig 4B shows that the junction region between the NACHT and LRR domains (dashed box) undergoes correlation pattern changes upon NEK7 binding to inactive NLRP3. This region includes the HD2 motif (a part of the NACHT domain) and the acidic loop (AL). NEK7 binding alters the AL’s correlation pattern with the NACHT domain, inducing a anti-correlation between them, and such anti-correlation is a feature in the active state’s DCCM profile. Additionally, we measured the loop structure content (defined as percentage of residues that from “coil” measure by VMD), and showed that NEK7 binding reduces loop formation in the HD2 motif while increasing it in AL (S3 Fig). These structural shifts render both motifs more similar to their conformations in activated NLRP3. Actually, we noticed that a large portion of the AL is unresolved in both PDBs 6NPY and 8EJ4. The missing regions were modeled using AlphaFold2 for our simulations (S4 Fig), and we noted the AL region is structurally translocated in PDB 6NPY versus 8EJ4. Since this AL region is highly variable in NLRs and has been implicated as a mediator of inactive NLRP3 oligomerization [20], the observation of translocation likely suggests that the structural states of AL has implications in NLRP3 inflammasome activation. Such translocation in PDB 6NPY versus 8EJR is likely induced during the oligomerization process.

Nevertheless, in our monomeric NLRP3 simulations, we also observed the structural state changes of the AL. The AL correlation changes observed in our simulations may thus prime the conformational transition of inactive NLRP3 toward the active state. By analyzing the RMSDs of the AL and HD2 motifs during simulations Fig 4C, we demonstrate that NEK7 binding stabilizes the HD2 motifs in inactive NLRP3, reducing the stability disparity between AL and HD2. This likely contributes to the enhanced correlation of AL with the NACHT domain. **Therefore, NEK7 binding reshapes inactive NLRP3’s dynamics to resemble the active state, an effect partially mediated by the AL and HD2 motifs.**

### 2.4. NEK7 redistributes the intermediate states in the conformational transition landscape of monomeric NLRP3

**Fig 5:**
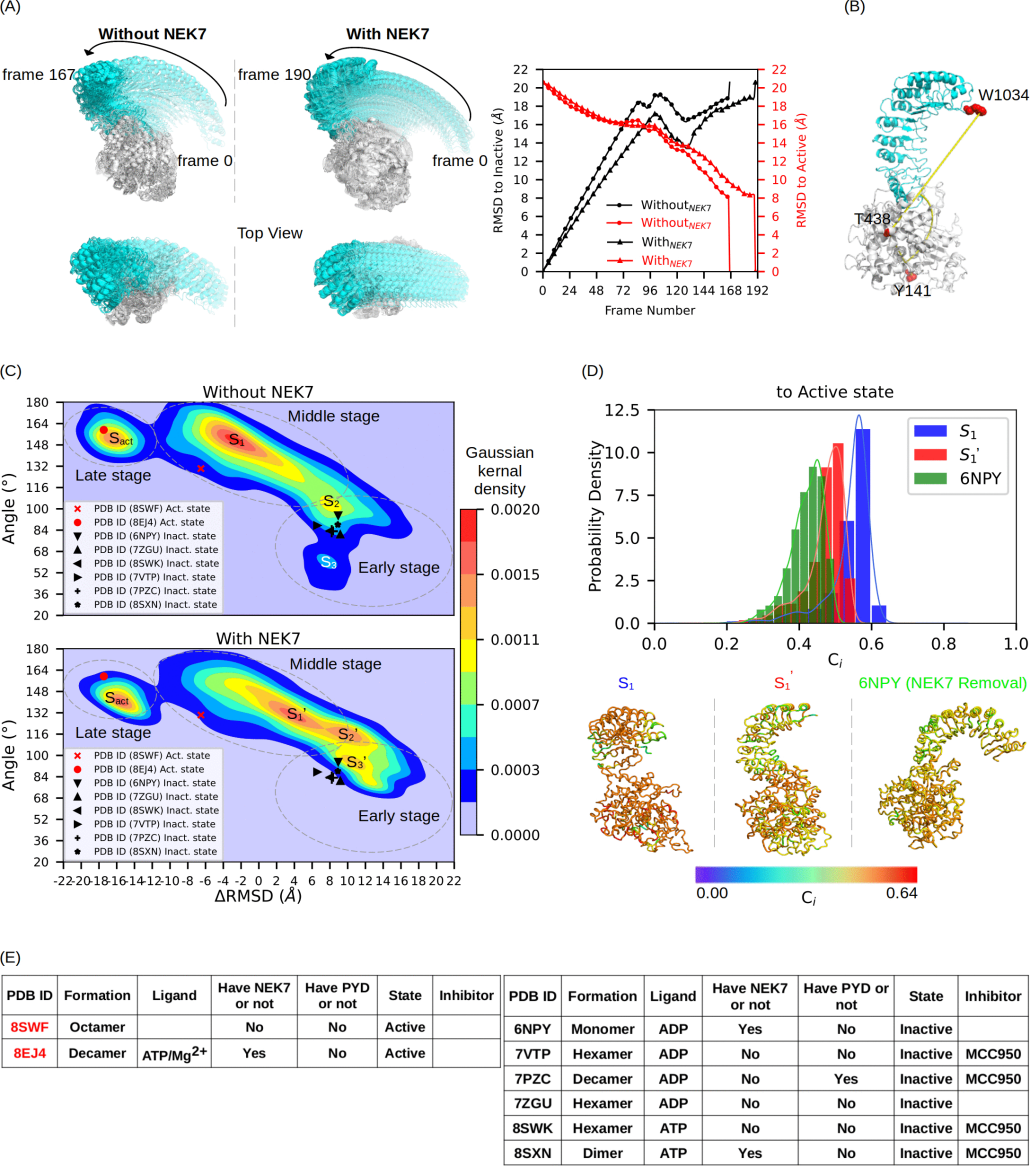
Effects of NEK7 on the conformational transition of monomeric NLRP3. **(A)** NMA-generated transition paths from the inactive-to-active NLRP3 monomer. Path structures were aligned to the FISNA-NBD-HD1 domain, with the WHD-HD2-AL-LRR domain gradually becoming more opaque as it approaches the active state conformation. RMSD values relative to the inactive (frame zero) and active NLRP3 conformations are plotted. **(B)** Definition of the Y141-T438-W1034 angle metric, which quantifies the rotational movement of the WHD-HD2-AL-LRR domain relative to the FISNA-NBD-HD1 domain. **(C)** Projections of MD samplings along the transition path onto a 2D space defined by ΔRMSD (x-axis) and the Y141-T438-W1034 angle (y-axis). The inactive-to-active transition is conceptually divided into early, middle, and late stages. Metastable states (S1/S1’, S2/S2’, S3/S3’, Sact) are identified as free energy minima on the landscape. Cryo-EM structures of human NLRP3 resolved under different conditions are mapped to the landscape. **(D)** PRS-calculated allosteric potentials of intermediate states S1, S1’ and inactive state (PDB 6NPY) structures toward the active state conformation. **(E)** Summary of Cryo-EM structures of human NLRP3 resolved under different conditions, with their locations on the transition landscape (panel C) indicated.

After demonstrating NEK7’s impact on the inactive NLRP3 monomer, we simulated the inactive-to-active conformational transition to explore NEK7’s role in intermediate states. To facilitate sampling, we first generated transition paths using normal mode analysis (NMA), then initiated unbiased conventional MD runs from these path structures to sample the transition conformational space and construct the landscape. Our previous work has demonstrated this strategy’s effectiveness for exploring multi-domain protein conformational transitions [21]. The NMA-generated transition paths connecting inactive and active NLRP3 monomers (Fig 5A) showed non-linear progression, evidenced by multiple minima in RMSD (to inactive) curves near the active state. This non-linearity likely reflects the concerted motion of NLRP3’s multiple domains during transition. Both paths followed a two-stage process: initial up-left LRR domain swing followed by LRR rotation relative to the NACHT domain. Notably, NEK7 altered the transition path, increasing the number of intermediate structures from 167 to 190 and slowing progression toward the active state in late transition stages.

To explore conformational space, we initiated hundreds of 60-ns unbiased MD simulations from path structures. These samplings, combined with end-state MD data, were projected onto two metrics for landscape construction: (1) ΔRMSD (RMSD_*toActive*_ - RMSD_*toInactive*_) measuring relative proximity to end states, and (2) a Y141-T438-W1034 angle (Fig 5B) capturing WHD-HD2-LRR-FISNA-NBD-HD1 rotation during transition [1,3,19]. The resulting landscape (Fig 5C) showed four metastable states in both NEK7-bound and unbound cases, though their positions differed. We divided the transition into early, middle, and late stages (each spanning ∼1/3 of ΔRMSD range). NEK7 clearly reshaped the landscape in early and middle stages. Without NEK7, early-stage samplings (S3 state) were dispersed, while NEK7 binding concentrated these samplings into a more productive S3’ state closer to the middle stage. However, in the middle stage, NEK7 appeared to impede progress by stabilizing the S1’ state farther from activation than the NEK7-free S1 state. To further confirm the impeding effect, we calculated the allosteric potentials of S1’ and S1 state structures towards the active state conformation using the perturbation response scanning (PRS) method [22]. PRS recorded the conformational displacement of the starting structure in response to random perturbation acted on each residue. By comparing the displacement with that required to transfer the starting structure to a target structure, a residue’s allosteric potential toward the target structure, $C_{i}$, was obtained. This $C_{i}$ values is defined as the Pearson coefficient between perturbation-induced conformational displacement with the starting-to-end structural displacement, with higher $C_{i}$ means greater allosteric potentials. In Fig 5D, S1 state conformations exhibited $C_{i}$ values centered at 0.6, higher than S1’ state values (∼0.5). Structural mapping showed that the NEK7-free S1 state had elevated $C_{i}$ values in both the NACHT and LRR domains, while the NEK7-bound S1’ state showed reduced $C_{i}$, particularly in the LRR domain (reducing its allosteric potential). The inactive monomer (PDB 6NPY) had even lower $C_{i}$ (∼0.4), attesting to its non-production dynamics towards active state conformation. Therefore, the NEK7-bound S1’ state is inferior to the NEK7-free S1 state in terms of allosteric potential in transition to the activated state conformation.

Notably, in both cases, the active states (S*_act_*) remained isolated by high energy barriers, suggesting oligomer assembly is required to complete the transition. This oligomer impact can also be inferred by the locations of different NRLP3 Cryo-EM structures that are resolved in various oligomeric states after mapping into our landscape. The two active state structures (8SWF-octamer and 8EJ4-decamer) occupy distinct positions, while six inactive structures cluster in the early transition stage. This broad distributions of these Cryo-EM structures in the landscape implies that, the oligomeric state, along with small molecules such as ADP, ATP and inhibitor etc are also affecting the conformational dynamics of monomeric NLRP3 (Fig 5E). In summary, NEK7 exerts stage-dependent regulation on monomeric NLRP3: it restricts non-productive conformations in early transition stages but stabilizes intermediate states farther from activation in middle stages. Importantly, regardless of NEK7 presence, monomeric NLRP3 cannot overcome the high energy barriers to reach fully activated conformations, underscoring the role of oligomer assembly in completing the transition process.

### 2.5. Verification of NEK7’s trapping effect on NLRP3 in the S1’ state

**Fig 6:**
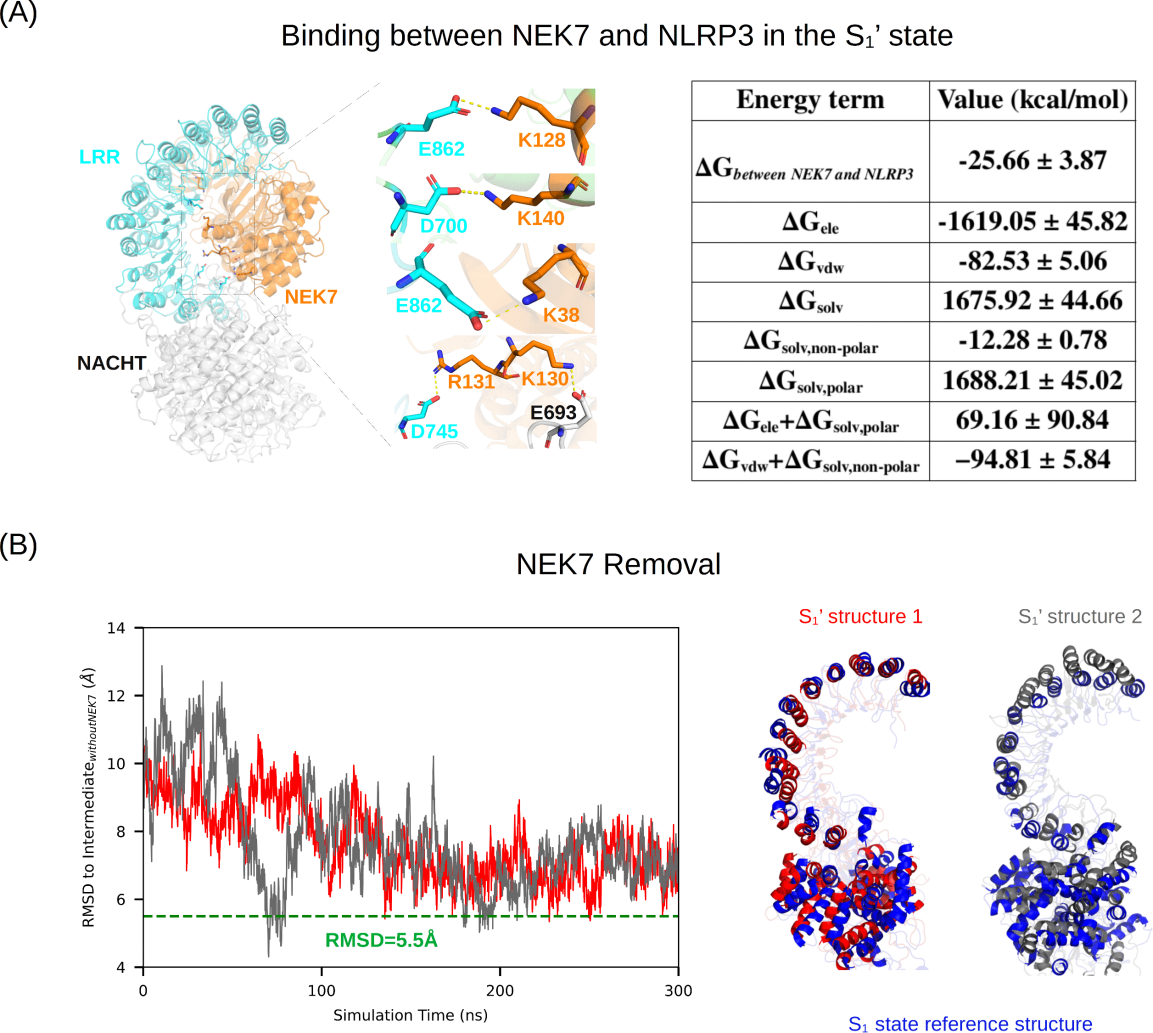
Trapping effects of NEK7 on S1’ state structures of NLRP3. **(A)** Example salt bridge between NEK7 (orange) and NLRP3 in the S1’ state, with MM/GBSA-calculated binding free energy (ΔG) between NEK7 and NLRP3 based on MD trajectories starting from twelve S1’ state structures. **(B)** RMSD trajectories of two S1’ structures relative to an S1 reference structure during 300 ns MD simulations (NEK7 removed prior to simulation). The green dashed line at RMSD = 5.5 Å was drawn to guide interpretation. Overlaid structures show convergence to the S1 state (blue) at the end of the simulation.

We demonstrated that NEK7 stabilizes an intermediate state (S1’ in Fig 5C) located farther from the active conformation. To confirm that NEK7 actively traps NLRP3 in this S1’ state, we selected representative S1’ conformations and performed MD simulations. The MM/GBSA calculations were performed to quantify the binding affinity between NEK7 and NLRP3 in the S1’ state. The resultant binding free energy (ΔG) of approximately –25 kcal/mol indicates a strong interaction, which supports our hypothesis that NEK7 can trap the NLRP3 monomer in this specific conformational state (Fig 6A). Furthermore, the energy decomposition analysis was conducted to elucidate the nature of this binding. The electrostatic interaction term ($\Delta G_{\text{ele}}$) is favorable (−1619 kcal/mol), reflecting strong electrostatic complementarity between NEK7 and NLRP3. However, the polar solvation term ($\Delta G_{\text{solv,polar}}$) is comparably large and positive (+1688 kcal/mol), indicating that desolvation of the charged protein–protein interface incurs a substantial energetic penalty. Consequently, the net electrostatic contribution ($\Delta G_{\text{ele}}+\Delta G_{\text{solv,polar}}$) is unfavorable (+69 kcal/mol). This suggests that while long-range electrostatic steering may facilitate initial encounter, the formation of the bound complex entails a significant desolvation cost. The shape/hydrophobic complementarity, reflected by the sum of the van der Waals term ($\Delta G_{\text{vdw}}$) and the nonpolar solvation term ($\Delta G_{\text{solv,non-polar}}$), is favorable at approximately −95 kcal/mol. Together with other energetic components, this makes the overall binding free energy to a spontaneous value of about −25 kcal/mol. In the NEK7-NLRP3 bound state, visual inspection identified key salt bridges that may stabilize the complex. Moreover, we conducted 300-ns atomistic MD simulations on two NEK7-free S1’ state structures by removing NEK7. Trajectory analysis showed transitions toward the S1 state, evidenced by decreasing RMSD values (converging to ∼5.5 Å relative to an S1 reference structure; Fig 6B). Structural overlays of relaxed conformations further confirmed convergence to S1-like states. Therefore, NEK7 traps NLRP3 in the inhibitory S1’ state via stable binding, while NEK7 removal alleviates this blockade, facilitating progression along the activation pathway.

## 3. Discussion

Our MD simulations showed that the isolated NLRP3 monomer exhibits different dynamic behaviors in its two end states: the inactive state is highly flexible, while the active state is stable, characterized by low RMSF regardless of NEK7 presence (Fig 4A). While low RMSF in the NEK7-bound active NLRP3 might be expected, as NEK7 could presumably stabilize the LRR domain, we found that this explanation is insufficient because the NEK7-free active monomer also shows comparably low RMSF. This low RMSF is also unlikely to be caused by ATP in the NACHT domain, as the LRR domain, which is distant from the NACHT domain, also exhibits low RMSF (Fig 4A). In fact, our cross-correlation analysis (Fig 4B) suggests a plausible explanation: in the active NLRP3 monomer, the interfacial regions between the LRR and NACHT domains are more tightly correlated than in the inactive monomer. This tighter correlation may restrict the relative motion between these two domains, resulting in the stability of the active NLRP3 monomer.

We demonstrated that inactive NLRP3 can adopt multiple conformations, whereas the activated state represents a predetermined, stable structural endpoint. Such structural features of the end state NLRP3 are reflected in the available Cryo-EM structures. While five inactive-state oligomers have been resolved under various conditions (PDB 7VTP, 7PZC, 7ZGU, 8SWK, and 8SXN), only one confirmed activated oligomeric state exists (PDB 8EJ4), along with the recently reported open octamer structure (see structural summary in Fig 5E). The inherent flexibility of the inactive monomer likely facilitates its ability to form different oligomeric states in response to diverse stimuli. We propose that this conformational flexibility of the inactive state, coupled with the predestinated stable active state, helps explain NLRP3’s diverse inflammasome activation mechanisms. Beyond canonical activation pathways involving potassium efflux [23], various non-canonical activation mechanisms exist, such as cold exposure-induced activation of NLRP3 mutants that depends on calcium (Ca^2+^) influx rather than the K^+^ efflux [24]. We argue that the inactive state’s structural adaptability enables broad responsiveness to different stimuli, while the stable active conformation represents a converged end point, ensuring consistent downstream cytokine production regardless of the activation pathway.

Our findings suggest that while NEK7 binding does not substantially reduce the overall conformational flexibility of inactive NLRP3, it reshapes its dynamics to resemble the activated state (Fig 4). This minimal structural impact but profound dynamic influence suggests that NEK7 plays a role in priming the early-stage conformational transition of inactive NLRP3 monomers. Our analysis of the NEK7-inactive NLRP3 interface supports this role: the binding interface is dynamic, as evidenced by rapidly exchanging hydrogen-bonding residues (Fig 3C). If the binding interface were fixed, such strong binding would likely hinder the conformational transition of the inactive monomer.

By combining the end state equilibrium simulations and the transition process simulation, we showed a stage-dependent role of NEK7 in the transition of monomeric NLRP3. NEK7 plays an important role in the early stage of the transition in terms of reshaping the dynamics, and in the middle stage of the transition, NEK7 redistributes the intermediate states in the conformation transition landscape. In the middle stage, NEK7 relocates the intermediate states further from the activated state, exhibiting an impeding effect. Interestingly, we showed that NEK7 has negligible impacts on the late stage transition (e.g., reaching the actual activated state), highlighting the assembly-assistance in finalizing the conformational transition. The irreverence of NEK7 on the final inflammasome disk formation may explain the molecular basis of NEK7-independent NLRP3 activation (LRR-deleted NLRP3’activation) [25], namely, NEK7 can be dispensable for the active disk formation. Actually, in the disk, it is the NACHT domain that nucleates the disk [3] to form the PYD-ASC filament required for cytokine production, supporting the dispensable role of NEK7 in the active disk formation.

Our sampled landscapes with and without NEK7, together with the locations of Cryo-EM inactive structures on these landscapes, suggest an alternative mechanism for NEK7-mediated disassembly of inactive NLRP3 oligomers. The current model, based on indirect structural evidence (PDB 6NPY and 7PZC), proposes that NEK7 binds the LRR domain and sterically clashes with an adjacent NLRP3 monomer in the cage, thereby promoting disassembly by competing for LRR-mediated interfaces [2]. However, direct structural evidence, such as an intermediate oligomeric state with NEK7 bound, remains lacking. Our landscape provides a dynamic perspective: in the absence of NEK7, inactive NLRP3 structures lie within the flexible S2–S3 region, whereas in the presence of NEK7, they are displaced to the rim of the stable states (Fig 5C). This shift indicates that NEK7 binding induces a significant conformational change in the inactive NLRP3 monomer, a change less pronounced without NEK7. Thus, NEK7’s ability to reshape the monomer’s conformational state offers a plausible mechanism for its role in oligomer disassembly. Furthermore, we infer that different inactive NLRP3 oligomers are similarly sensitive to NEK7’s disassembly-promoting effect, as the monomeric NLRP3 subunits within them are structurally similar and they are closely clustered on the landscape.

A limitation of our transition simulations is the omission of ATP/ADP and Mg^2+^. To assess whether this omission biases our sampled landscape, we performed the following analysis: First, we simulated gain-of-function (GOF) mutations within the ATP-binding pocket (E306K and L413F) [9]. As shown in S5 Fig, 200 ns atomistic simulations of the major state structures (S1 and S1’) with these mutations did not shift sampling into new basins, and conformations remained near the original major states. Second, we directly tested the effect of adding ATP and Mg^2+^ to two consecutive frames from the NMA-generated transition path. As shown in S6 Fig, while the presence of cofactors stabilized the sampling, the overall sampled regions on the ΔRMSD–angle plane largely overlapped between simulations with and without cofactors. Local structural analyses (S6C and S6D Figs) showed that cofactors induced moderate RMSD changes (2–3 Å) in the ATP pocket and slightly stabilized the WHD hinge region, but did not alter the global conformational landscape. These results suggest that although cofactors influence local fluctuations and sampling stability, the major states we identified are robust to their omission.

Additionally, our choice to focus on monomeric NLRP3 also bears limitations. This is because the conformational landscape of NLRP3, and thereby the structural activation mechanism of the NLRP3 inflammasome, is modulated by multiple factors that interplay with each other, and yet remains to be fully explored. Besides the nucleotides mentioned above, other factors such as the NLRP3 inhibitor MCC950 and the constructs used for cryo-EM studies (full-length versus PYD-deleted) can also lead to different oligomeric states of NLRP3 (as summarized in Fig 5C). However, we would like to emphasize that, among these factors, the NEK7 cofactor has a well-documented role in NLRP3 activation, as its presence is confirmed in both the inactive (PDB: 6NPY) and active (PDB: 8EJ4) states of NLRP3 [1,3]. Our study was not designed to propose NEK7 as the primary driver of NLRP3 conformational change. Instead, we sought to explore the possible effects of NEK7 on the conformational transition of the NLRP3 monomer from its inactive to active state, a dynamic process that is elusive to capture with experimental structural methods alone.

Explaining NLRP3 activation requires consideration of oligomeric states and the complex cellular environment. Our findings, albeit in silico, directly support and provide a mechanistic basis for the necessity of oligomerization in NLRP3 activation. Two key observations support this conclusion: (1) monomeric NLRP3 samples metastable states distant from the active state conformation, regardless of NEK7 presence; and (2) our transition landscape suggests a high energy barrier separating intermediate states from the active state conformation. These findings align with recent work by Xu et al. [19], demonstrating that the inactive-to-active transition in monomeric NLRP3 (even with NEK7) is energetically unfavorable. Notably, their study shows that neighboring NLRP3 monomers convert this uphill process into a thermodynamically favorable downhill transition. This compelling evidence for assembly-driven activation corroborates both our data and Cryo-EM structures of intermediate NLRP3 octamer [10]. Additionally, there are biochemical studies suggest that purified NLRP3 proteins exist in oligomeric states [26], highlighting the importance of oligomeric states in inflammasome activation. Our modeling results complement existing static structural snapshots by providing a mechanistic basis for the necessity of oligomerization in NLRP3 activation.

## 4. Conclusions

In this study, we employed multi-scale molecular simulations to investigate NEK7’s impact on large-scale conformational transitions of monomeric NLRP3 during inflammasome activation. Our methodology integrates protein dynamics and free energy landscape analysis, enabling mapping of NLRP3’s inactive-to-active transition and assessment of NEK7’s role from dynamic and energetic perspectives. We demonstrate a stage-dependent regulatory mechanism for NEK7, different from the simplistic binary view of its function as inhibitory or activating. Specifically, at the early stage of transition, NEK7 promotes transition initiation by reshaping inactive NLRP3 dynamics. And at the middle stage, NEK7 modestly impedes transition by stabilizing a less-productive intermediate state. At the late stage, NEK7’s influence diminishes, as inherent energy barriers isolate the active conformation regardless of NEK7 presence. These findings underscore the necessity of oligomeric assembly to complete activation, consistent with biochemical evidence of pre-assembled NLRP3 complexes and Cryo-EM structures of activation intermediates. The late stage negligible impact of NEK7 may explain the NEK7-independent non-canonical activation mechanisms of NLRP3 inflammasome. Our work suggests a multi-layered activation mechanism where oligomer-level assembly and monomeric conformational changes should be coupled, providing new mechanistic insights into this physiologically important macromolecular process.

## 5. Materials and methods

### 5.1. Monomeric NLRP3 structure preparations

Several inactive-state Cryo-EM structures of NLRP3 have been resolved: one as a monomer (PDB: 6NPY [1]) and others as oligomers (PDBs: 7ALV [27], 7VTP [28], and 7PZC [20]) and dimers (PDB 8SXN [10]). The NLRP3 conformations in these structures are broadly similar, with C_*α*_ root-mean-square deviations (RMSDs) between 6NPY and the oligomeric states of 2.8 Å, 2.0 Å, 2.7 Å, and 1.6 Årespectively (measured by Pymol’s align command with cycles = 0). We selected 6NPY over 8SXN as the starting structure for simulations of the inactive NLRP3 state for several reasons. First, PDB 6NPY was solved as a monomeric NLRP3–NEK7 complex, whereas PDB 8SXN is part of a dimeric assembly. Since our simulations aim to study the monomeric conformational landscape, the monomeric context of 6NPY aligns more directly with our simulation setup. Second, we noted that residues 537–628 in PDB 6NPY are differently positioned compared to those in PDB 8SXN. Because this region is located within the acidic loop motif that is known to be highly variable [20], the different positioning of these residues in PDB 6NPY may represent a unique structural state specific to monomeric NLRP3, and thus makes 6NPY advantageous over PDB 8SXN for monomeric NLRP3 simulations. Selecting PDB 8SXN over PDB 6NPY might offer the advantage of providing a more complete NEK7 structure, as NEK7 in PDB 8SXN has both its N- and C-lobes resolved, whereas in PDB 6NPY only the C-lobe is resolved. However, in both structures, inactive NLRP3 interacts exclusively with the C-lobe of NEK7; the additional N-lobe resolved in 8SXN does not participate in the NLRP3–NEK7 interface (S7 Fig). Therefore, the missing N-lobe in 6NPY should not compromise the representation of the NLRP3–NEK7 interaction relevant to our study. These considerations led us to select PDB 6NPY over PDB 8SXN as the starting structure for simulations of inactive NLRP3.

For the activated-state conformation, we used the disc-shaped NLRP3 inflammasome (PDB: 8EJ4 [3]). The inactive state system (based on PDB: 6NPY) contained ADP. Although a Mg^2+^ ion was not resolved in this structure, we added one based on the experimental conditions and by referencing a related NLRP3 NACHT domain structure with both ADP and Mg^2+^ bound (PDB: 8ETR). The active state system (based on PDB: 8EJ4) contained the ATP analog ATPγS and Mg^2+^. For our simulations, we replaced ATPγS with ATP. Both PDBs 6NPY and 8EJ4 include NEK7, but the resolved NEK7 constructs in these two PDB differ: 6NPY contains residues 113–300 (C-lobe only, N-lobe was not resolved), while 8EJ4 includes residues 20–297 (covering both N- and C-lobes). We used the full-length NEK7 construct for consistency in all our simulations. And this full-length NEK7 structure is from homology modeling. Notably, the C-lobe Cα RMSD between the NEK7 constructs in 6NPY and 8EJ4 is 1.86 Å, indicating that no conformational changes in NEK7 upon binding to distinct NLRP3 states. To maintain consistency, we used the longer 20–297 NEK7 construct for both states. Residues 20–112 of NEK7 (missing in 6NPY) were added using AlphaFold-predicted coordinates for full-length human NEK7. Both PDBs 6NPY and 8EJ4 also have missing NLRP3 residues. These gaps were filled by transferring coordinates from AlphaFold-predicted human NLRP3 (UniProt ID: Q96P20). The PYD of NLRP3 was excluded from simulations (see Fig 1A). The total numbers of amino acids of monomer NLRP3ΔPYD and NEK7 are 894 and 278, respectively. The MD system including waters and ions contain roughly 350,000 atoms on average.

In summary, PDBs 6NPY and 8EJ4 served as starting structures for the inactive and activated NEK7-bound monomeric NLRP3 conformations, respectively. NEK7 was removed from both systems, yielding two unbound states. Conventional MD simulations were performed for 3×1 μs on each system.

### 5.2. Conventional molecular dynamics (cMD) simulations

The proteins were described by the CHARMM36 force field [29] with the SPC/E water model [30]. The simulation box dimensions were 147×147× 147 Å^3^, and NaCl was added to maintain a 0.15 M salt concentration, approximating a typical cytosolic environment. Periodic boundary conditions were applied in all directions. The system was first energy-minimized using the steepest descent method with step size of 0.001 nm and a convergence criterion of maximum force < 500.0 kJ/mol/nm^2^. The minimized system was then heated to 300 K over 100 ps in the NPT ensemble, with pressure equilibrated at 1 bar. During heating, protein heavy atoms were restrained with harmonic potentials (force constant = 1000 kJ/mol/nm^2^). Three independent 1 *mu*s-long production runs were performed for each system in the NPT ensemble at 300 K. Non-bonded interactions used a 1.2 nm cutoff, and the MD time step was 2 fs. The LINCS algorithm constrained hydrogen bonds, while the PME (Particle Mesh Ewald) method handled long-range electrostatics. Simulations were conducted using GROMACS 2020.4 [31].

### 5.3. Normal mode analysis (NMA)-based transition path generation

The conformational difference between the inactive and active states of NLRP3 is large, characterized by a rigid rotation of ∼90° of the WHD-HD2-LRR domain relative to the FISNA-NBD-HD1 domain [2,3]. Modeling such complex conformational transitions is challenging due to their reliance on collective motions across multiple domains. Normal Mode Analysis (NMA) is well-suited for identifying these collective motions in stable protein structures. We employed the NMA-based structural morphing technique proposed by López-Blanco *et al* [32] to generate transition paths between the inactive (PDB 6NPY) and activated (PDB 8EJ4) states of NLRP3. This method calculates structural displacements using low-frequency normal modes, with weights optimized to transition the system toward the target structure (here, the activated state). Displacements were accepted if they exceeded an RMSD-based threshold while ensuring progression toward the target conformation. Iterative application of this process yielded morphed structures resembling the target state [32]. Simulations were performed for two scenarios: (1) transition from PDB 6NPY to PDB 8EJ4 without NEK7 and (2) with NEK7 retained. All systems excluded ADP/ATP and Mg^2+^ ions; only NLRP3 and NEK7 (if present) were included, represented by their C_*α*_ atoms. Torsion angles served as internal coordinates for NMA. Transition paths were sampled at 0.5 Å RMSD intervals. The iMOD program [32] produced two paths: 1) Path 1 (without NEK7) that contains 167 intermediate structures, and 2) Path 2 (with NEK7) that contains 190 intermediate structures.

### 5.4. Transition path structure-seeded conventional MD

Starting from each structure in the NMA-generated transition path, a 60 ns production MD simulation in the NPT ensemble at 300 K was performed without any restraints to expand conformational sampling. The simulation system contained no ADP/ATP/Mg^2+^. This decision was made because the transition process involves ATP hydrolysis and significant conformational changes at the nucleotide-binding site. It is impractical to assign a specific nucleotide state (ADP vs. ATP) to a continuous conformational transition. Since our primary goal was to explore the specific role of NEK7, omitting the ligands from both the presence and absence of NEK7 simulations effectively minimize for ligands’ effect. For Path 2 (with NEK7), the NLRP3-NEK7 complex was solvated in a water box containing 0.15 M NaCl. The total conventional MD (cMD) length was 190×60 ns = 11.4 μs. For Path 1 (without NEK7), only NLRP3 was simulated, with a total cMD length of 167×60 ns = 10.02 μs. To assess the potential impact of the omission of cofactors, we performed two sets of simulations. One set introduced gain-of-function (GOF) mutations E306K and L413F [9] around the ATP binding pocket into the major state structures to explore how such perturbations might alter these structures. For the S1 state (without NEK7) and the S1’ state (with NEK7), each structure was selected and subjected to 200 ns MD simulations, both with and without the GOF mutations. Another attempt involved adding back the cofactors (ATP and Mg^2+^) to the NMA-generated path structures and then running MD simulations to assess how the presence of cofactors affects the sampling relative to runs without cofactors. We selected the 56th and 57th frames from the NEK7-free transition path and ran 200 ns MD simulations with and without cofactors. All simulations followed the protocol described above and are summarized in S1 Table.

### 5.5. Perturbation response scanning (PRS)

The PRS algorithm evaluates the allosteric signature of a structure by applying random forces to individual residues and recording conformational displacements [33]. Conformational displacement (ΔR) caused by a perturbation (Δ𝐅) on residue *i* can be quantified using linear response theory (LRT), assuming the variance-covariance matrix (**C**) is known [22]:


$$\begin{array}{c} \Delta R=\langle R\rangle_{1}-\langle R\rangle_{0}\sim \frac{1}{kT}𝐂\Delta 𝐅 \end{array}$$ (1)

To assess the allosteric potential of an initial structure toward a target structure, we correlate perturbation-induced displacements with the displacement between the initial and target structures [22]:


$$\begin{array}{c} C_{i}=\frac{\sum_{j=1}^{N}[(\Delta R_{j}{)}^{i}-(\overset{―}{\Delta R}{)}^{i}](\Delta S_{j}-\overset{―}{\Delta S})}{(N-1)\sigma_{R}\sigma_{S}} \end{array}$$ (2)

where *N* is total number of residues, $(\Delta R_{j}{)}^{i}$ is the displacement of residue *j* during perturbation of residue *i*, $\Delta S_{j}$ is displacement of residue *j* between initial and target structures. $\sigma_{R}$ and $\sigma_{S}$ are standard deviations of $(\Delta R_{j}{)}^{i}$ and $\Delta S_{j}$, respectively. Higher $C_{i}$ values indicate residues that, when perturbed, induce displacements that are more correlated with the wanted displacement, suggesting enhanced allosteric activity. In this study, after identifying metastable states along the conformational transition pathway of monomeric NLRP3, we extracted representative structures from these states and performed PRS to evaluate their allosteric potential toward the activated state. Each representative structure underwent 40 ns of conventional MD to compute the variance-covariance matrix **C**. PRS calculations were executed using MDM-TASK-Web [34,35], applying 100 perturbations per residue.

### 5.6. Analysis

Conventional analyses including RMSD, RMSF, angle, and distance measurements were performed using GROMACS analysis tools and CPPTRAJ [36]. Time-dependent intermolecular hydrogen bond counts between NEK7 and NLRP3 was determined using the *hbond* command from CPPTRAJ. Specifically, for each NLRP3 residue, the total number of hydrogen bonds it formed with NEK7 at each frame was counted, as was plotted versus simulation time. Atom density projection onto the XY-plane was done using the *gmx densmap* tool based on the conventional MD trajectories. For each case, the accumulated 3×1 μs trajectories were first aligned to the LRR domain of PDB 6NPY with pre-fixed orientation prior to gmx densmap calculations. The output density profile was then plotted using the Matplotlib module. The DCCM of NLRP3 was calculated by using the Dynamic Cross-Correlation tool in MD-TASK [35] using the accumulated 3×1 μs trajectories for each case. To construct the free energy landscape, all MD samplings were projected onto a 2D plane that has the ΔRMSD (x-axis) and the Y141-T438-W1034 angle (y-axis) as axes (see definition in main-text). The density on the 2D plane was then estimated via Gaussian kernels using the gaussian_kde function from the scipy python library. The MM/GBSA binding free energy between NEK7 and NLRP3 in the S1’ state was calculated using the gmx_MMPBSA [37] tool under ionic strength of 0.15 M, based on 300 ns MD trajectories of the NEK7-NLRP3 complex. Data visualization was implemented with the Matplotlib Python library, while structural renderings were generated using PyMOL and ChimeraX. Scripts used in this project are available at: https://github.com/bsu233/bslab/tree/main/2025-NLRP3. Simulation trajectories are deposited in a publicly available Zenodo repository at https://doi.org/10.5281/zenodo.19394630.

## Supporting information

S1 Fig Hydrogen bonds between NEK7 and NLRP3 in the inactive and active states. (A) Illustration of NEK7-NLRP3 binding in inactive (PDB 6NPY) and active (PDB 8EJ4) states of NLRP3. (B-C) Specific hydrogen bonds formed between NEK7 and NLRP3 in the two end states.
(PNG)

S2 FigRadius of gyration (R_*g*_) of NLRP3 in the end states.The brown, magenta, and blue bars represent data from three parallel 1μs MD trajectory, with representative conformation of NLRP3 taken from the largest bin. We showed that the R_*g*_ range of the two inactive systems are larger than that of the two active systems, indicating the conformational flexibility of the inactive NLRP3 monomers. Additionally, in the inactive-without-NEK7 system, the maximum value of the R_*g*_ around 33–36 Å is close the R_*g*_ values reported by El-Sayed et al [PMID: 36173167].(PNG)

S3 FigLoop structure in key NLRP3 motifs from conventional MD simulations of end states.To assess the structural states of key NLRP3 motifs, we calculated secondary structure elements from three independent 1-μs MD simulations. For each motif, we report the percentage of residues forming loop structures as a means to assess the structural states. Each motif is represented by three bars corresponding to the three independent simulation replicates.(PNG)

S4 Fig Acidic loop (AL) structure in PDB 8EJ4 and in 6NPY. The usage of AlphaFold2 to predict the missing residues in the AL regions in these two PDBs were shown.
(JPG)

S5 Fig Effects of GOF mutation on major state structures. Two GOF mutations E306K and L413F were introduced onto the most populated state structures (S1 and S1’ states of the with and without NEKY landscape), and run 200 ns atomistic MD simulation. The selected structure was denoted as a single dot, and the resulting sampled conformations were plotted as scattered dots. (A) The landscape without mutations. (B) The landscape of two GOF mutations.
(PNG)

S6 FigImpact of cofactor omission on the transition simulation.(a) Two consecutive frames (56th and 57th) from the NMA-generated transition path in the absence of NEK7 were selected. For each structure, 200 ns all-atom MD simulations were performed in the absence and presence of cofactors (ATP and Mg^2+^), respectively. (b) Projection of the sampling onto the ΔRMSD–angle 2D plane to assess overlap between simulations with and without cofactors. (c) Assessment of cofactor impact on the ATP binding pocket. Residues within 5 Å of ADP in PDB 6NPY were selected, and their time-dependent RMSD relative to the starting structure was plotted. (d) Assessment of cofactor impact on the WHD hinge region. The WHD region from PDB 6NPY is shown, with its time-dependent RMSD plotted.(PNG)

S7 Fig NEK7 binding modes with inactive NLRP3 in PDB 6NPY versus PDB 8SXN. (PNG)

S1 Table All molecular dynamics simulations performed in the present study. (PNG)
