# Supplementary figures and images for: Stage-dependent role of NEK7 in the inactive-to-active conformational transition of NLRP3 monomer
Source: PLoS Comput Biol. 2026 Jun 12;22(6):e1014405. doi: 10.1371/journal.pcbi.1014405 (PMC13274922; doi:10.1371/journal.pcbi.1014405)

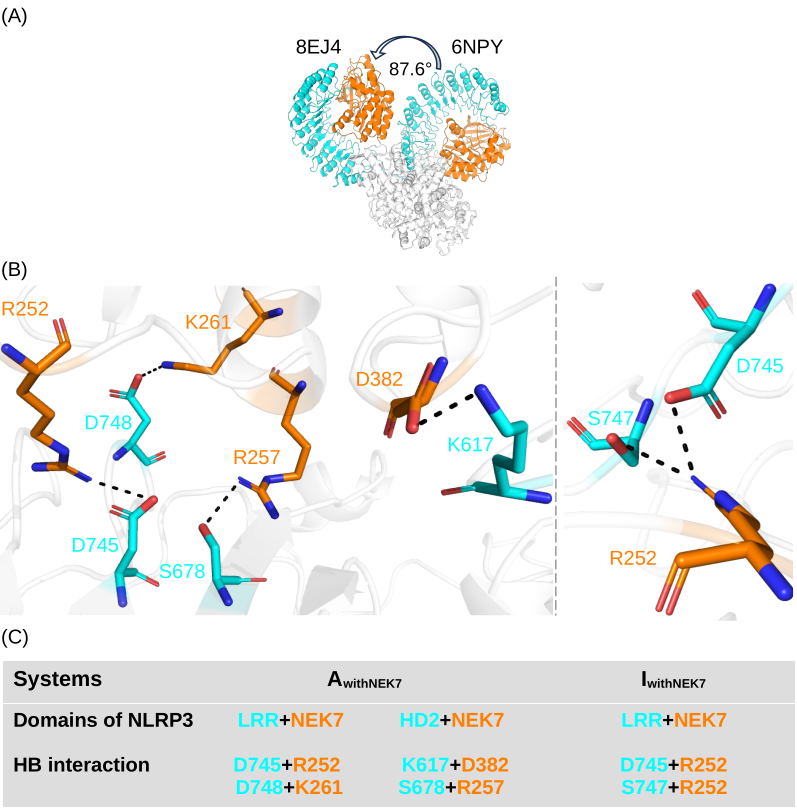

Supplement: S1 Fig — (A) Illustration of NEK7-NLRP3 binding in inactive (PDB 6NPY) and active (PDB 8EJ4) states of NLRP3. (B-C) Specific hydrogen bonds formed between NEK7 and NLRP3 in the two end states. (PNG) [file pcbi.1014405.s001.png]

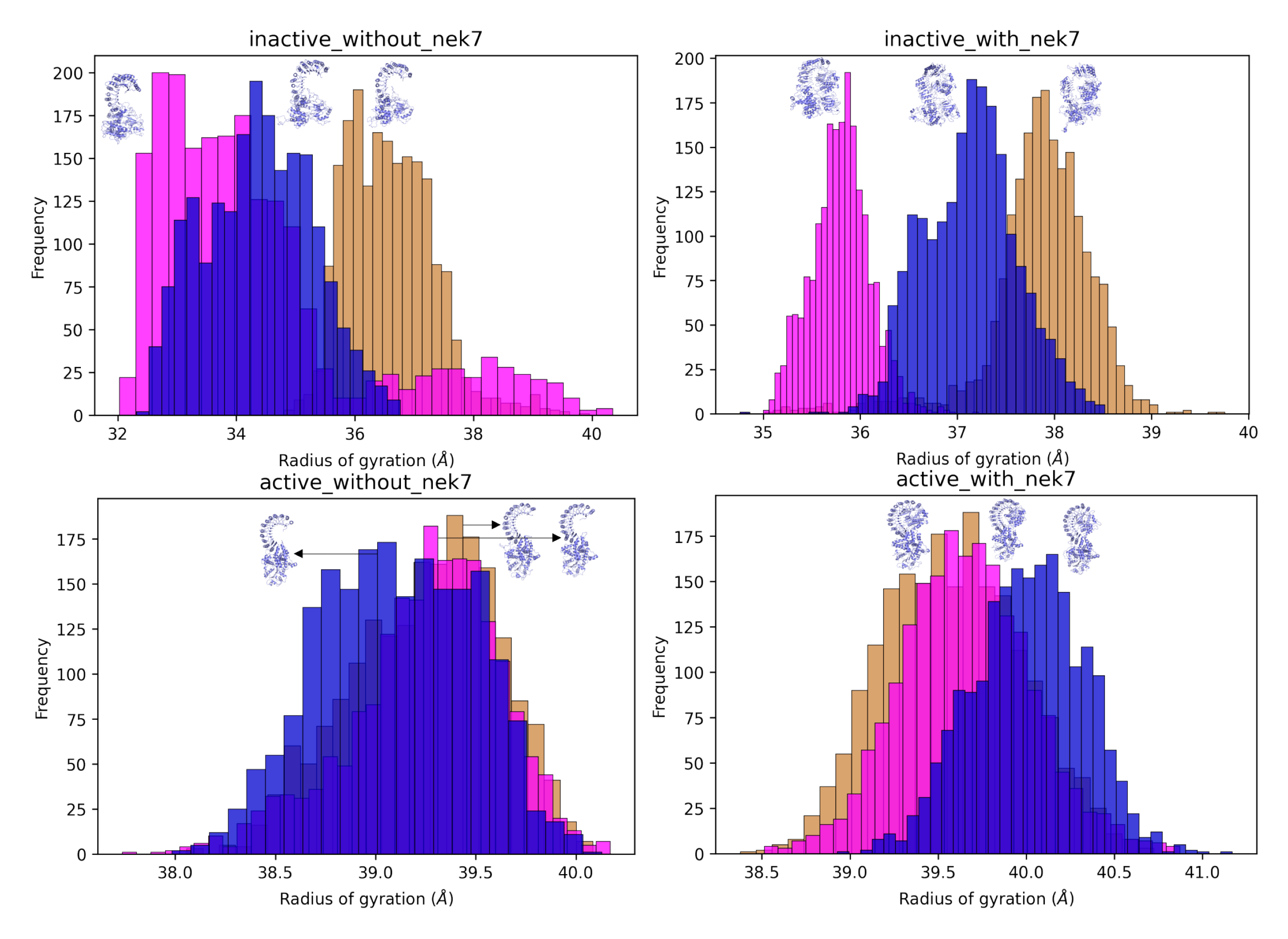

Supplement: S2 Fig — The brown, magenta, and blue bars represent data from three parallel 1μs MD trajectory, with representative conformation of NLRP3 taken from the largest bin. We showed that the Rg range of the two inactive systems are larger than that of the two active systems, indicating the conformational flexibility of the inactive NLRP3 monomers. Additionally, in the inactive-without-NEK7 system, the maximum value of the Rg around 33–36 Å is close the Rg values reported by El-Sayed et al [PMID: 36173167]. (PNG) [file pcbi.1014405.s002.png]

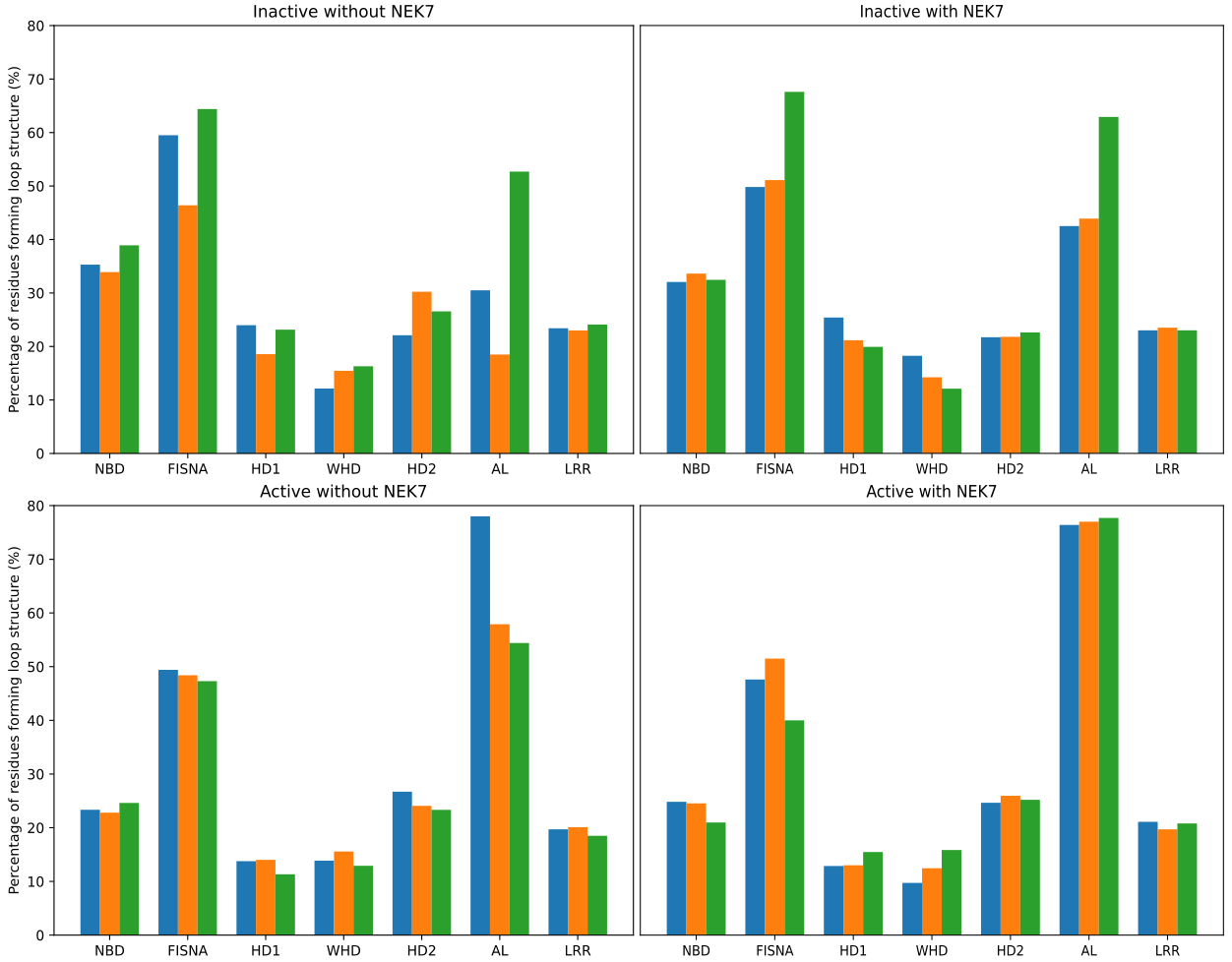

Supplement: S3 Fig — To assess the structural states of key NLRP3 motifs, we calculated secondary structure elements from three independent 1-μs MD simulations. For each motif, we report the percentage of residues forming loop structures as a means to assess the structural states. Each motif is represented by three bars corresponding to the three independent simulation replicates. (PNG) [file pcbi.1014405.s003.png]

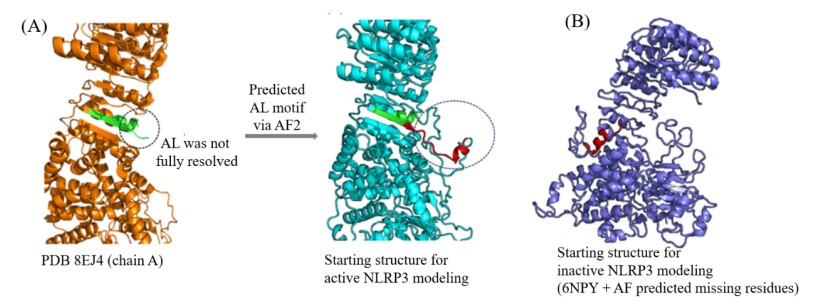

Supplement: S4 Fig — The usage of AlphaFold2 to predict the missing residues in the AL regions in these two PDBs were shown. (JPG) [file pcbi.1014405.s004.jpg]

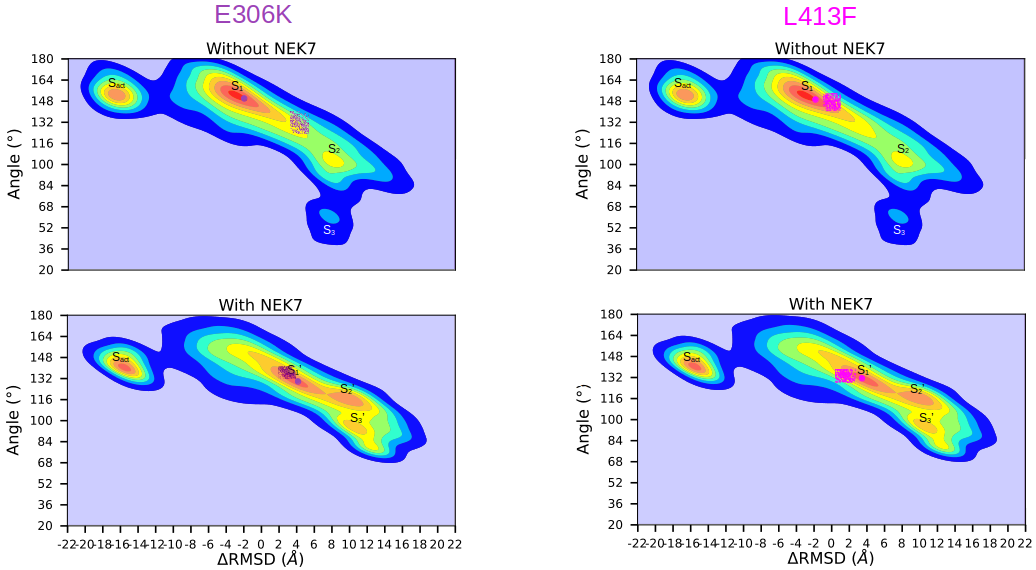

Supplement: S5 Fig — Two GOF mutations E306K and L413F were introduced onto the most populated state structures (S1 and S1’ states of the with and without NEKY landscape), and run 200 ns atomistic MD simulation. The selected structure was denoted as a single dot, and the resulting sampled conformations were plotted as scattered dots. (A) The landscape without mutations. (B) The landscape of two GOF mutations. (PNG) [file pcbi.1014405.s005.png]

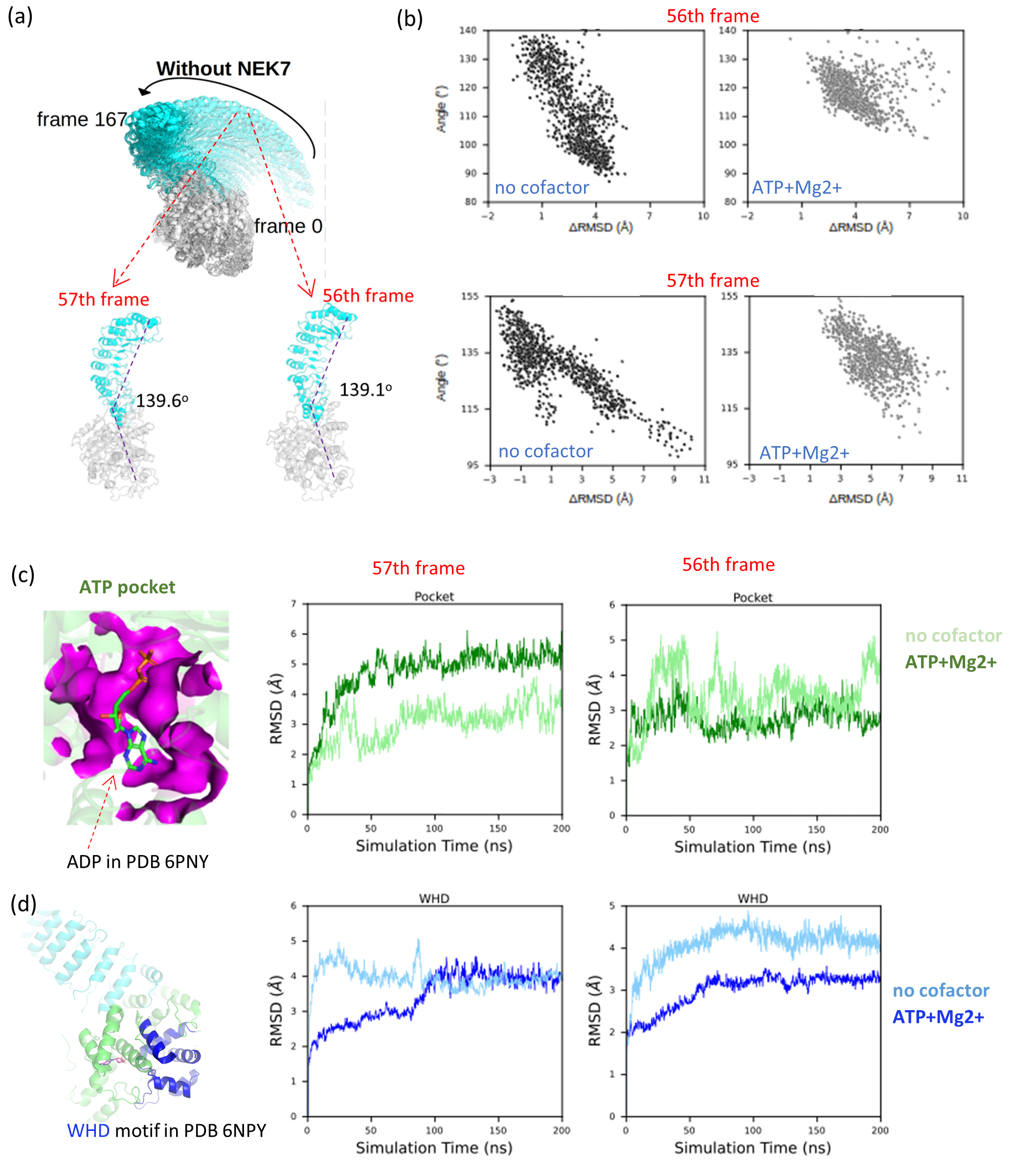

Supplement: S6 Fig — (a) Two consecutive frames (56th and 57th) from the NMA-generated transition path in the absence of NEK7 were selected. For each structure, 200 ns all-atom MD simulations were performed in the absence and presence of cofactors (ATP and Mg2+), respectively. (b) Projection of the sampling onto the ΔRMSD–angle 2D plane to assess overlap between simulations with and without cofactors. (c) Assessment of cofactor impact on the ATP binding pocket. Residues within 5 Å of ADP in PDB 6NPY were selected, and their time-dependent RMSD relative to the starting structure was plotted. (d) Assessment of cofactor impact on the WHD hinge region. The WHD region from PDB 6NPY is shown, with its time-dependent RMSD plotted. (PNG) [file pcbi.1014405.s006.png]

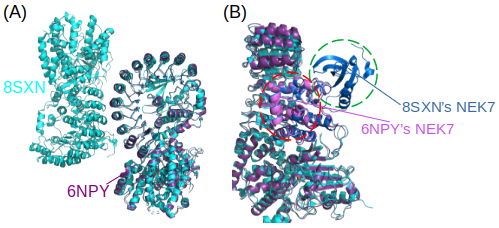

Supplement: S7 Fig — (PNG) [file pcbi.1014405.s007.png]

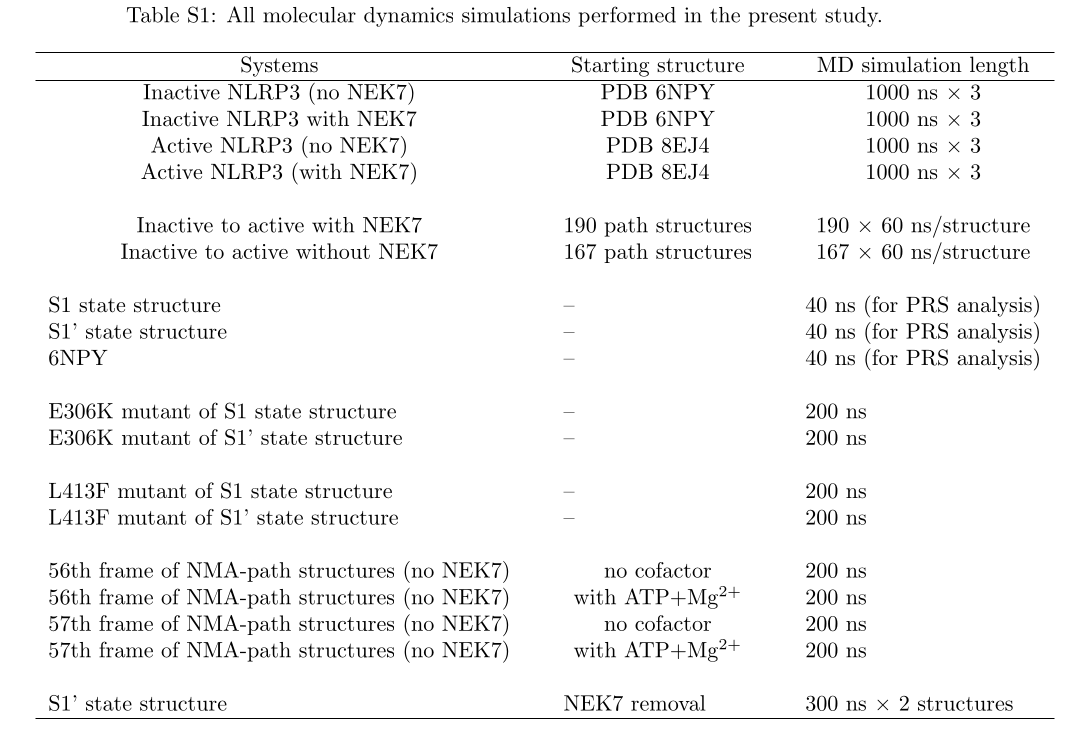

Supplement: S1 Table — (PNG) [file pcbi.1014405.s008.png]
