# Supplementary material for: Stage-dependent role of NEK7 in the inactive-to-active conformational transition of NLRP3 monomer
Source: PLoS Comput Biol. 2026 Jun 12;22(6):e1014405. doi: 10.1371/journal.pcbi.1014405 (PMC13274922; doi:10.1371/journal.pcbi.1014405)
Supplement: Attachment — Submitted filename: point-to-point-response.pdf [file pcbi.1014405.s010.pdf]

0.1 Peer Reviewer #1:

This manuscript uses state-of-the-art computational approaches to characterize how the NEK7 co-factor affects the inactive-to-active conformational transition of monomeric NLRP3. The main claims are that NEK7 has a stage-dependent role: (i) at early stages NEK7 reshapes dynamics of inactive NLRP3 making it more active-like, (ii) at middle stages NEK7 stabilizing a less-productive intermediate state, and (iii) at late stages NEK7 has negligible effect because a high energy barrier separates the active conformation (implying oligomerization is required to complete activation). The study integrates multiple simulation modalities and attempts to map Cryo-EM structures into the computed landscape. Results are interesting and plausible and address an important mechanistic question for NLRP3 inflammasome regulation.

1. *Role of nucleotides (ATP/ADP) during transition simulations: Although incorporating the effect of ATP hydrolysis into MD simulations is nontrivial, run simulations on models bearing gain-of-function mutations around the ATP binding pocket to test whether nucleotide-site perturbations alter the transition landscape. It would provide valuable insights into NLRP3 activation.*

   Response: We thank the reviewer for this insightful comment, which highlights an important limitation of our study. We fully agree that incorporating nucleotides (ATP/ADP and Mg^{2+}) into transition simulations is non-trivial, and we deliberately omitted them to keep the enhanced sampling tractable. As the reviewer notes, an alternative approach to probe the influence of the nucleotide is to simulate gain-of-function (GOF) mutations in that region. We have therefore carried out the suggested simulations and report the results below.

   We selected two GOF mutations in the NLRP3 ATP-binding pocket: E306K (a charge-reversing mutation) and L413F (a hydrophobic-to-aromatic substitution) [1]. To assess whether these nucleotide-site perturbations alter the transition landscape, we performed 200 ns atomistic simulations of the major state structures (each structure from S1 and S1' states) from the landscapes with and without NEK7. The resulting samplings were projected onto the original reaction coordinates (ΔRMSD and angle) to visualize any shifts relative to the unperturbed states.

   As shown in Fig. X1, the E306K mutation induced noticeable conformational changes in the S1 structure (starting from ΔRMSD $\sim$0 Å and angle $\sim$148°, the sampled conformations spread to ΔRMSD $\sim$ 4-6 Å and angle around 131°). While these changes are substantial, the sampled conformations remained near the rim of the S1 basin. In the presence of NEK7, the S1' state was largely unaffected by E306K. The L413F mutation resulted in only minimal deviations for both S1 and S1'. Thus, within this simulation timeframe, the GOF mutations did not drive the system into distinct alternative states. These results, together with our later results shown in Fig. X4, suggest that the omission of ATP/ADP likely does not fundamentally alter the identity of the major states we identified. We have added a discussion paragraph to reflect these points. **See tag R1R2 on Page 31, Line 689**

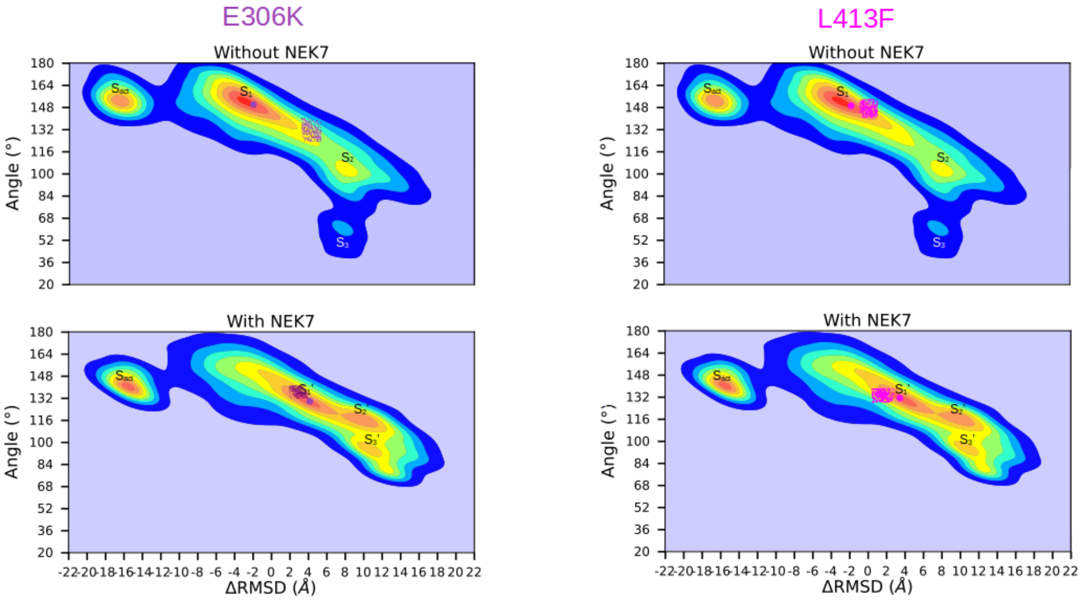

Figure X1: Two GOF mutations E306K and L413F [1] were introduced onto the most populated state structures (S1 and S1' states of the with and without NEKY landscape), and were subject to 200 ns atomistic MD simulation. The selected structure was denoted as a single dot, and the resulting sampled conformations were plotted as scattered dots.

2. *NLRP3 oligomerization features and NEK7-mediated disassembly: The authors note that NEK7 contributes to NLRP3 activation and that NLRP3 oligomerization is pivotal for the conformational transition to the active state. Both inactive cage and intermediate oligomeric state were reported previously—whose formation and conformational shifts may be NEK7-independent—suggest a possible role for NEK7 in oligomer disassembly, thereby enabling assembly of the canonical inflammasome disk. The authors might therefore provide mechanistic hypotheses on how NEK7 mediates dissociation and indicate which reported oligomeric forms are predicted by their landscape to be most/least susceptible to NEK7-mediated dissociation.*

   Response: We thank the reviewer for this insightful comment, which has provided us a unique perspective to relate our work to NEK7's role in mediating inactive oligomer disassembly.

   Before addressing the comment, we first note that in our original Figure 5C the ΔRMSD coordinates of the Cryo-EM NLRP3 structures on the landscape need to be corrected, because we mixed up their amino acid numbering when calculating the RMSD; the NLRP3 sequences in these structures contain slightly different numbers of residues. We have recalculated the ΔRMSD using the consensus NLRP3 region common to all structures and updated Figure 5C (see Fig. X2), while keeping all other elements unchanged. The interpretation of this figure remains the same.

   In the updated Figure 5C (Fig. X2), all inactive NLRP3 structures are now clustered closely together, corroborating their overall structural similarity.

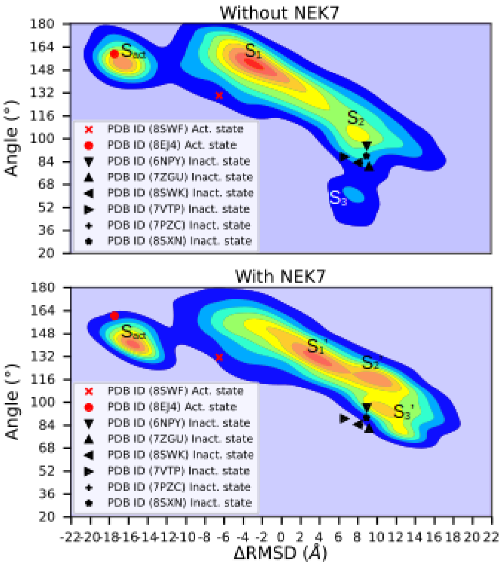

Figure X2: The corrected Figure 5C, in which the ΔRMSD coordinates of Cryo-EM NLRP3 structures on the landscape have been updated.

Returning to the reviewer's comment, as the reviewer has noted, the general notion that NEK7 plays a role in mediating inactive NLRP3 oligomer disassembly is based on indirect structural evidence from PDB 6NPY (inactive NLRP3 monomer bound to NEK7) and PDB 7PZC (NLRP3 cage formed without NEK7) [2]. Specifically, in the monomer–NEK7 complex, NEK7 binds to the LRR domain of NLRP3. In the cage, adjacent NLRP3 monomers also interact via their LRR domains. When the monomer–NEK7 complex is placed in the cage context, a steric clash occurs between NEK7 and the LRR domain of the neighboring NLRP3 monomer. This has led to the proposal that NEK7 promotes cage disassembly by competing for LRR-mediated interfaces, as summarized by Fu *et al* [3].

As the reviewer has implied, although this mechanism is appealing, direct structural evidence (e.g., an intermediate oligomeric state with NEK7 bound) is still lacking. Our results may provide a more dynamic perspective on NEK7's role in mediating inactive NLRP3 oligomer disassembly. This can be inferred from the location of the inactive NLRP3 structures relative to the sampled region in our landscape.

In the absence of NEK7, these inactive NLRP3 structures lie within the conformational space spanning S2 and S3 (Fig. X2), showing that inactive NLRP3, free of NEK7, is flexible and can sample broad conformational space. In the presence of NEK7, all of the reported inactive oligomeric forms are located outside the stable states (e.g., at the rim of the sampled conformational space). This suggests that upon NEK7 binding, a significant conformational change of the inactive NLRP3 monomer should occur, whereas such a change is less obvious in the NEK7-free case (Fig. X2). NEK7's ability to reshape the inactive NLRP3 monomer's conformational state is thus a plausible mechanism to explain NEK7's role in mediating oligomer disassembly.

As for predicting which oligomeric NLRP3 form is most susceptible to NEK7's effect, because the different inactive oligomeric structures cluster tightly in the landscape due to their overall structural similarity, we speculate that they are likely all similarly sensitive to NEK7-mediated disassembly.

We have incorporated these points into the revised manuscript. **See tag R1Q2 on Page 31, Line 674**

3. *The choice of initial inactive-state model (PDB 6NPY vs 8SXN) The authors used PDB 6NPY as the starting structure for the inactive NLRP3 simulations rather than PDB 8SXN, justifying this choice on the grounds that 8SXN represents a dimeric, pharmacologically inhibited state complexed with MCC950. This rationale requires clarification: 6NPY itself was solved in the presence of ADP, MgCl2 and MCC950 (0.3 mM ADP, 5 mM MgCl2, 0.3 mM MCC950; Humayun Sharif et al., Nature 570:338–343, 2019). Moreover, 8SXN resolves both the N- and C- lobes of NEK7 whereas 6NPY lacks part of the N-lobe. Because these differences (such as oligomeric context and resolved regions) may influence MD and NEK7 interactions, the authors should provide a more plausible justification for choosing 6NPY as the inactive-state model. Or additional simulations (e.g., using 8SXN-derived starting structures or testing the presence/absence of MCC950) to demonstrate that the main conclusions are not dependent on the choice of initial PDB.*

   Response: We thank the reviewer for this thoughtful comment and for pointing out our error regarding the presence of MCC950 in PDB 6NPY.

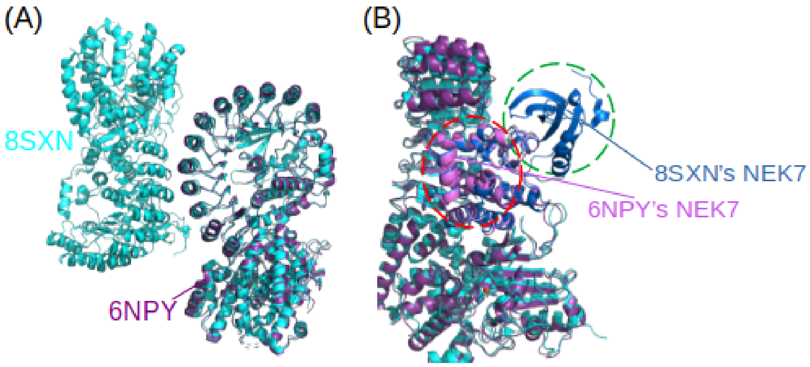

Figure X3: Comparison of inactive NLRP3 structures from PDB 6NPY and PDB 8SXN.

In our original manuscript, we stated that we chose PDB 6NPY as the starting structure because it appeared to be inhibitor-free (as no MCC950 was present in the PDB file), whereas PDB 8SXN was solved with MCC950. As the reviewer correctly notes, during the experimental procedure of PDB 6NPY determination, MCC950 was also present. Our original justification was therefore not well-supported, and we thank the reviewer for this correction.

Instead, we would like to add the following arguments to more strongly justify our choice of 6NPY: First, PDB 6NPY was solved as a monomeric NLRP3–NEK7 complex, whereas PDB 8SXN is part of a dimeric assembly. Since our simulations aim to study the monomeric conformational landscape, the monomeric context of 6NPY aligns more directly with our simulation setup. Second, we noted that residues 537–628 in PDB 6NPY are differently positioned compared to those in PDB 8SXN. Because this region is located within the acidic loop motif that is known to be highly variable [4], the different positioning of these residues in PDB 6NPY may represent a unique structural state specific to monomeric NLRP3, and thus makes 6NPY advantageous over PDB 8SXN for monomeric NLRP3 simulations [4].

As the reviewer has suggested, selecting PDB 8SXN over PDB 6NPY might offer the advantage of providing a more complete NEK7 structure, as NEK7 in PDB 8SXN has both its N- and C-lobes resolved, whereas in PDB 6NPY only the C-lobe is resolved. However,

in both structures, inactive NLRP3 interacts exclusively with the C-lobe of NEK7; the additional N-lobe resolved in 8SXN does not participate in the NLRP3–NEK7 interface (Fig. X3). Therefore, the missing N-lobe in 6NPY should not compromise the representation of the NLRP3–NEK7 interaction relevant to our study. These considerations led us to select PDB 6NPY over PDB 8SXN as the starting structure for simulations of inactive NLRP3. We have added these arguments to better rationalize our choice of PDB 6NPY **See tag R1Q3 on Page 32, Line 758**

Minor:

1. *Correction may be needed: NLRP3 does not contain the "N-terminal CARD domain" (Introduction)*

   Response: We appreciate these corrections, and have revised accordingly.

2. *Minor specific typos found, for example: "NKE7" → "NEK7" (Conclusions). "the diddle stage" → "the middle stage" (Conclusions). "an high" → "a high" (multiple places). "outpout density" → "output density" (Methods 5.6).*

   Response: Thanks for the careful reading of our manuscript. We have double-checked and corrected these typos.

0.2 Peer Reviewer #2:

This interesting work uses molecular dynamics simulations to assess the effect of kinase NEK7 binding to NLRP3 protein on NLRP3's ability to adopt an active state conformation. The authors find that NEK7 binding biases the NLRP3 towards more active-like states initially, but that a significant barrier remains regarding assumption of the completely formed active state. This finding is consistent with previous simulation studies, in particular that of Xu et al. (ref 16) but also provides new insight into possible intermediate states. The methods employed appear soundly executed and simulations suitably analyzed and interpreted. The results should be of interest to PLoS Comp Biol readers and I recommend the work for publication after consideration of the following remarks:

1. *The study makes a couple of significant assumptions. The first is the study of solely monomer, as NLRP3 is generally thought to exist as oligomer in inactive and active forms. Cooperative action of the aggregate is likely to play a significant role in activation. However, interesting mechanical insights can still be derived by a reductionist study here of the monomer. In the Introduction, the discussion of experimental evidence for the (i) existence and importance of monomer and (ii) the latest thinking regarding the role of NEK7 needs to be expanded with more references.*

   Response: We thank the reviewer for this insightful comment.

   The two aspects the reviewer suggested we expand upon are highly valuable and will help better place our computational work within the broader context of NLRP3 mechanism studies. Interestingly, we found a new study published in 2025 that supports the existence and physiological importance of the NLRP3 monomer, along with additional studies that reinforce the critical role of the NEK7–NLRP3 interaction in inflammasome activation.

   Specifically, work by Borši'c *et al* [5] shows that the membrane can promote NLRP3 clustering to allow inflammasome activation even in the absence of canonical activators. Their model presumes that inactive NLRP3 can exist either in cage-like structures or as monomers. While the end point of activation is a disk-like oligomer, the starting state of NLRP3 (the inactive form) can vary, ranging from monomers to dimers to larger assemblies.

   Regarding the critical role of the NEK7–NLRP3 interaction in inflammasome activation, three recent studies provide compelling evidence. Zhang *et al* [6] and Jin *et al* [7] both showed that disrupting the NEK7–NLRP3 interaction—via metabolic isocyanic acid or a small-molecule inhibitor, respectively—inhibits inflammasome activation. Conversely, Xu *et al* [8] demonstrated that phosphorylated NEK7 enhances NEK7–NLRP3 binding and accentuates inflammasome activation. Collectively, these studies underscore the importance of the NEK7–NLRP3 interaction in NLRP3 inflammasome activation.

   We would also like to offer an additional perspective to highlight the relevance of our computational work. Although it is well established that NLRP3 operates in oligomeric forms under physiological conditions, from a biophysical standpoint these oligomers are composed of individual NLRP3 monomers. The monomer serves as the basic building block of the oligomeric inflammasome; therefore, understanding the internal dynamics of the NLRP3 monomer is directly relevant to the structural activation mechanism of the higher-order inflammasome complex.

   We have incorporated these points into the Introduction section of the revised manuscript.

   See tag R2Q1 on Page 15, Line 367

2. *The second assumption is the omission of cofactor for the transition simulations (but not the end point simulations). The state of the cofactor is known to be very important in activation of NLRP3. The authors also argue that omission of cofactor from both NEK7-bound and unbound forms mitigates the assumption of no cofactor, which does not seem a strong assumption. The authors also attempt to explore for a couple of snapshots the effect of this on RMSD and RMSF (quite low sensitivity measures). The two snapshots appear to have the domains at different degrees of rotation of LRR with respect to NACHT domain and thus provides something of a test (what are the angle values and time points of the samples?). What is the effect of this omission on the cofactor binding pocket residues and the adjacent domains which are very important to the hinge transition?*

   Response: We thank the reviewer for these insightful comments.

   Although our initial choice to omit cofactors in the transition simulations was justified to a certain extent (because ATP hydrolysis is coupled with NLRP3 conformational transitions, and it is nontrivial to assign which nucleotide (ATP or ADP) corresponds to specific transition frames), we agree with the reviewer that a more in-depth analysis of the potential bias that cofactor omission could introduce is both necessary and helpful for strengthening our findings.

   During the review process, Reviewer 1 also suggested a useful means to assess this impact by introducing gain-of-function (GOF) mutations around the ATP pocket. As shown in Fig. X1, GOF mutations in most cases did not cause significant conformational changes in structures selected from the major states of our landscape. These results serve as one piece of evidence that omitting the cofactor has limited impact on our conclusions.

   Returning to the reviewer's comment on our attempt to assess the effect of cofactor omission, we agree that RMSD and RMSF are relatively low-sensitivity measures and were not an ideal choice (Figure S5 in our original manuscript). PS: We regret to note that in the original Figure S5 legend, we stated that the two structures were selected from the S1' state of the NEK7-bound case; in fact, they were randomly selected from the NMA-generated transition path of the NEK7-free case. We apologize for this mistake.

   Here, we re-selected two consecutive frames (56th and 57th) from the NMA-generated transition path and added back ATP and Mg^{2+} to the NACHT domain (Fig. X4a). We then performed 200 ns all-atom MD simulations for each structure, both with and without cofactors. Projecting the sampling onto the ΔRMSD–angle 2D plane (Fig. X4b), we observed that the sampling in the absence of cofactors was less concentrated than in the presence of cofactors, suggesting that cofactors exert a stabilizing effect on the sampling. Importantly, the sampled conformations with and without cofactors largely overlapped for both structures. Zooming in on specific structural motifs, we found that cofactors induced approximately 2–3 Å RMSD differences in the ATP pocket and slightly stabilized the WHD region, reducing its RMSD by about 2 Å (Fig. X4c–d). These data indicate that while cofactors can cause considerable local changes in important structural motifs within the NACHT domain, the overall major states sampled by the MD simulations were not significantly altered.

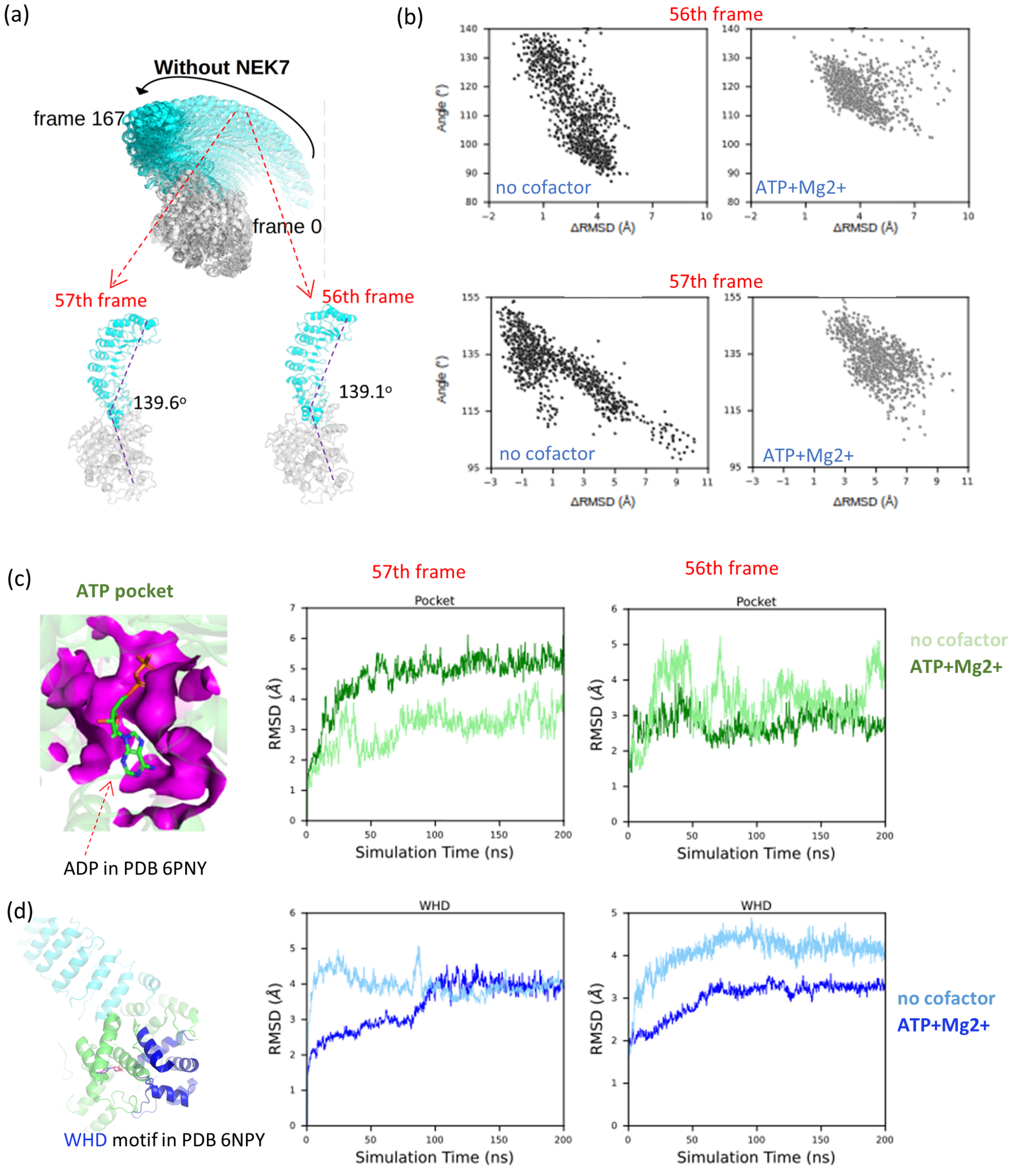

Figure X4: Assessing the potential impact of cofactor omission on the transition simulation. (a) Two consecutive frames (56th and 57th) from the NMA-generated transition path in the absence of NEK7 were selected. For each structure, 200 ns all-atom MD simulations were performed in the absence and presence of cofactors (ATP and Mg^{2+}), respectively. (b) Projection of the sampling onto the ΔRMSD–angle 2D plane to assess overlap between simulations with and without cofactors. (c) Assessment of cofactor impact on the ATP binding pocket. Residues within 5 Å of ADP in PDB 6NPY were selected, and their time-dependent RMSD relative to the starting structure was plotted. (d) Assessment of cofactor impact on the WHD hinge region. The WHD region from PDB 6NPY is shown, with its time-dependent RMSD plotted.

We have now added a Discussion section detailing our analysis of the potential impact of cofactor omission on our sampling, focusing on the newly added Fig. X1 and Fig. X4. **See tag R1R2 on Page 31, Line 689**

We appreciate the reviewers for their insightful comments.

3. *On a related note, it is not entirely clear why at the molecular level the active conformation has lower RMSFs and is more restricted ("intrinsically stable") than the inactive form. The authors call this observation "remarkable". Is this due to the presence of ATP to some degree? Some discussion of this would be useful.*

   Response: We thank the reviewer for this comment. The observation that the active NLRP3 monomer exhibits low RMSF, regardless of NEK7 binding, was also interesting to us. Initially, we thought that the low RMSF in the NEK7-bound case might be expected, as NEK7 could presumably stabilize the LRR domain. However, we found that this explanation is insufficient because the NEK7-free active monomer also shows comparably low RMSF. We therefore described this low-RMSF profile as an intrinsic feature of the active NLRP3 monomer and referred to the observation as "remarkable."

   The reviewer's comment prompted us to analyze this interesting phenomenon more deeply. After excluding the effect of NEK7 binding, we argue that ATP is also not the cause of low RMSF, because the LRR domain, which is far from the NACHT domain, also exhibits low RMSF (Fig. 4A). In fact, our cross-correlation analysis (Fig. 4B) suggests a plausible explanation: in the active NLRP3 monomer, the interfacial regions between the LRR and NACHT domains are more tightly correlated than in the inactive monomer. This tighter correlation may restrict the relative motion between these two domains, leading to the reduction in RMSF.

   We have briefly added this point as one possible explanation for the low RMSF of the active monomer in the revised manuscript. **See tag R2Q3 on Page 30, Line 626**

Minor:

1. *Why is 6NPY far from the sampled region in Fig. 5C and 5D? Were gradually reducing constraints used to carefully equilibrate the protein? A brief comment on the resolution of the cryo-EM structures may also be useful, given their resolution is not that high.*

   Response: We thank the reviewer for the careful reading. We double-checked and verified that the original Figure 5C had incorrect ΔRMSD coordinates for the Cryo-EM structures because we mismatched their amino acid numbering when calculating the RMSD; the NLRP3 sequences in these structures contain slightly different numbers of residues. We have now recalculated the ΔRMSD using the consensus NLRP3 region common to all structures and updated Figure 5C (see Fig. X2). In the corrected figure, NLRP3 from PDB 6NPY and the other inactive oligomeric states are located close to each other, and 6NPY is now positioned near the sampled region. We sincerely apologize for the mistake in our original Figure 5C.

2. *Fig. 3(B) – why is the x-axis showing 3 us and yet 3 replicate simulations appear to be shown? Was it 3x 3 us or 3x 1 us MD simulations?*

   Response: We thank the reviewer for pointing out the labeling error in Fig. 3B. We have verified that the x-axis should read 1 μs, and the three differently colored curves represent three independent 1 μs MD simulations. The label has been corrected accordingly.

3. *Section 2.3 - How is "loop" formation defined ?*

   Response: We thank the reviewer for this question. We assume the reviewer is referring to the following sentence in Section 2.3: "NEK7 binding reduces loop formation in the HD2 motif while increasing it in AL" (Fig. S3).

   In our analysis, a "loop" is defined based on secondary structure assignment performed in VMD. Specifically, each amino acid residue is classified into one of the following secondary

structure types: α-helix, 3-10 helix, Pi-helix, bridge, extended configuration and turn. Residues that do not belong to any of the above categories are assigned as coil. In our study, residues assigned as coil were considered to form a loop. We have briefly added the definition in the sentence. **See tag R2Qm3 on Page 22, Line 522**

4. *In places the language appears unduly emphatic, eg. "hallmark", "uniquely" and "reveal". These are predictions from MD simulations using models. Similarly the word "drive" is used in several places although this is conjecture rather than proven in the work.*

   Response: We thank the reviewer for this comment. We have revised the wording throughout the manuscript to better reflect the computational nature of our work and have toned down the language where appropriate.

5. *We note that while there clearly is electrostatic complementarity between NEK7 and NLRP3, the MMGBSA electrostatic terms of NEK7-NLRP3 largely cancel in Fig 6A; the van der Waals term is negative (the opposing nonpolar solvation term is not stated).*

   Response: We thank the reviewer for the comment. As suggested, we decomposed the MMGBSA free energy and present the individual components in a new panel (Fig. X5).

   The electrostatic interaction term (ΔG_{ele}) is favorable (-1619 kcal/mol), reflecting strong electrostatic complementarity between NEK7 and NLRP3. However, the polar solvation term ($\Delta G_{\text{solv,polar}}$) is comparably large and positive (+1688 kcal/mol), indicating that desolvation of the charged protein–protein interface incurs a substantial energetic penalty. Consequently, the net electrostatic contribution ($\Delta G_{\text{ele}} + \Delta G_{\text{solv,polar}}$) is unfavorable (+69 kcal/mol). This suggests that while long-range electrostatic steering may facilitate initial encounter, the formation of the bound complex entails a significant desolvation cost.

   The shape/hydrophobic complementarity, reflected by the sum of the van der Waals term (ΔG_{vdw}) and the nonpolar solvation term ($\Delta G_{\text{solv,non-polar}}$), is favorable at approximately -95 kcal/mol. Together with other energetic components, this makes the overall binding free energy to a spontaneous value of about -25 kcal/mol.

   We have revised Figure 6A and added a brief discussion of the energy terms in the manuscript. **See tag R2Qm5 on Page 28, Line 606**

Energy term	Value (kcal/mol)
$\Delta G_{\textit{between NEK7 and NLRP3}}$	-25.66 ± 3.87
ΔG_{ele}	-1619.05 ± 45.82
ΔG_{vdw}	-82.53 ± 5.06
ΔG_{solv}	1675.92 ± 44.66
$\Delta G_{solv,non\text{-}polar}$	-12.28 ± 0.78
$\Delta G_{solv,polar}$	1688.21 ± 45.02
$\Delta G_{ele}+\Delta G_{solv,polar}$	69.16 ± 90.84
$\Delta G_{vdw}+\Delta G_{solv,non\text{-}polar}$	–94.81 ± 5.84

Figure X5: Energy decomposition of the MM/GBSA results between NEK7 and NLRP3 corresponding to Figure 6A.

6. *"diddle" should be "middle" ; NKE not NEK in several places; Fig. 4 zoomed-in box is*

not obvious; "LINCS" not "LINC";Cite Lopez-Blanco et al. correctly from text;Covariance equation symbols missing

Response: Thanks for your careful reading of our manuscript. We have made changes accordingly.
