# Supplementary material for: Stage-dependent role of NEK7 in the inactive-to-active conformational transition of NLRP3 monomer
Source: PLoS Comput Biol. 2026 Jun 12;22(6):e1014405. doi: 10.1371/journal.pcbi.1014405 (PMC13274922; doi:10.1371/journal.pcbi.1014405)
Supplement: Peer review history

# Peer review: Decision Letter 0

**DOI:** 10.1371/journal.pcbi.1014405.r001

**Authors:** Dirk Walther

16 Feb 2026

PCOMPBIOL-D-25-02711

Stage-dependent role of NEK7 in the inactive-to-active conformational transition of NLRP3 monomer

PLOS Computational Biology

Dear Dr. Sun,

Thank you for submitting your manuscript to PLOS Computational Biology. After careful consideration, we feel that it has merit but may benefit from addressing the suggestions raised by the expert reviwers. Therefore, we invite you to submit a revised version of the manuscript that addresses the points raised during the review process.

Please submit your revised manuscript by Apr 18 2026 11:59PM. If you will need more time than this to complete your revisions, please reply to this message or contact the journal office at ploscompbiol@plos.org. When you're ready to submit your revision, log on to https://www.editorialmanager.com/pcompbiol/ and select the 'Submissions Needing Revision' folder to locate your manuscript file.

Please include the following items when submitting your revised manuscript:

* A letter that responds to each point raised by the editor and reviewer(s). You should upload this letter as a separate file labeled 'Response to Reviewers'. This file does not need to include responses to formatting updates and technical items listed in the 'Journal Requirements' section below.

* A marked-up copy of your manuscript that highlights changes made to the original version. You should upload this as a separate file labeled 'Revised Manuscript with Track Changes'.

* An unmarked version of your revised paper without tracked changes. You should upload this as a separate file labeled 'Manuscript'.

If you would like to make changes to your financial disclosure, competing interests statement, or data availability statement, please make these updates within the submission form at the time of resubmission. Guidelines for resubmitting your figure files are available below the reviewer comments at the end of this letter

We look forward to receiving your revised manuscript.

Kind regards,

Dirk Walther, Ph.D.

Section Editor

PLOS Computational Biology

Dirk Walther

Section Editor

PLOS Computational Biology

**Additional Editor Comments:**

Dear authors, thanks for your submission to PLoS Comp Biol. Your work has now been reviewed by two expert reviewers. Both found the study interesting and of merit, but made a number of relevant suggestions, which I hope can be addressed. I look forward to receiving your revised version. Best regards, Dirk Walther

**Journal Requirements:**

If the reviewer comments include a recommendation to cite specific previously published works, please review and evaluate these publications to determine whether they are relevant and should be cited. There is no requirement to cite these works unless the editor has indicated otherwise.

1) Please provide an Author Summary. This should appear in your manuscript between the Abstract (if applicable) and the Introduction, and should be 150-200 words long. The aim should be to make your findings accessible to a wide audience that includes both scientists and non-scientists. Sample summaries can be found on our website under Submission Guidelines:

https://journals.plos.org/ploscompbiol/s/submission-guidelines#loc-parts-of-a-submission

2) Please upload all main figures as separate Figure files in .tif or .eps format. For more information about how to convert and format your figure files please see our guidelines:

https://journals.plos.org/ploscompbiol/s/figures

3) We notice that your supplementary Figures are included in the manuscript file. Please remove them and upload them with the file type 'Supporting Information'. Please ensure that each Supporting Information file has a legend listed in the manuscript after the references list.

4) In the online submission form, you indicated that your data will be submitted to a repository upon acceptance. We strongly recommend all authors deposit their data before acceptance, as the process can be lengthy and hold up publication timelines. Please note that, though access restrictions are acceptable now, your entire minimal dataset will need to be made freely accessible if your manuscript is accepted for publication. This policy applies to all data except where public deposition would breach compliance with the protocol approved by your research ethics board. If you are unable to adhere to our open data policy, please kindly revise your statement to explain your reasoning and we will seek the editor's input on an exemption.

5) In the online submission form, you indicated that Simulation trajectories are available upon request.. All PLOS journals now require all data underlying the findings described in their manuscript to be freely available to other researchers, either

1. In a public repository

2. Within the manuscript itself

3. Uploaded as supplementary information.

This policy applies to all data except where public deposition would breach compliance with the protocol approved by your research ethics board. If your data cannot be made publicly available for ethical or legal reasons (e.g., public availability would compromise patient privacy), please explain your reasons by return email and your exemption request will be escalated to the editor for approval. Your exemption request will be handled independently and will not hold up the peer review process, but will need to be resolved should your manuscript be accepted for publication. One of the Editorial team will then be in touch if there are any issues.

6) Please amend your detailed Financial Disclosure statement. This is published with the article. It must therefore be completed in full sentences and contain the exact wording you wish to be published.

1) State the initials, alongside each funding source, of each author to receive each grant. For example: "This work was supported by the National Institutes of Health (####### to AM; ###### to CJ) and the National Science Foundation (###### to AM)."

2) State what role the funders took in the study. If the funders had no role in your study, please state: "The funders had no role in study design, data collection and analysis, decision to publish, or preparation of the manuscript."

3) If any authors received a salary from any of your funders, please state which authors and which funders..

If you did not receive any funding for this study, please simply state: u201cThe authors received no specific funding for this work.u201d

7)  Please ensure that the funders and grant numbers match between the Financial Disclosure field and the Funding Information tab in your submission form. Note that the funders must be provided in the same order in both places as well.

**Reviewers' comments:**

Reviewer's Responses to Questions

**Comments to the Authors:**

**Please note here if the review is uploaded as an attachment.**

Reviewer #1: This manuscript uses state-of-the-art computational approaches to characterize how the NEK7 co-factor affects the inactive-to-active conformational transition of monomeric NLRP3. The main claims are that NEK7 has a stage-dependent role: (i) at early stages NEK7 reshapes dynamics of inactive NLRP3 making it more active-like, (ii) at middle stages NEK7 stabilizing a less-productive intermediate state, and (iii) at late stages NEK7 has negligible effect because a high energy barrier separates the active conformation (implying oligomerization is required to complete activation).

The study integrates multiple simulation modalities and attempts to map Cryo-EM structures into the computed landscape. Results are interesting and plausible and address an important mechanistic question for NLRP3 inflammasome regulation.

Major comment 1 — Role of nucleotides (ATP/ADP) during transition simulations

Although incorporating the effect of ATP hydrolysis into MD simulations is nontrivial, run simulations on models bearing gain-of-function mutations around the ATP binding pocket to test whether nucleotide-site perturbations alter the transition landscape. It would provide valuable insights into NLRP3 activation.

Major comment 2 —NLRP3 oligomerization features and NEK7-mediated disassembly

The authors note that NEK7 contributes to NLRP3 activation and that NLRP3 oligomerization is pivotal for the conformational transition to the active state. Both inactive cage and intermediate oligomeric state were reported previously—whose formation and conformational shifts may be NEK7-independent—suggest a possible role for NEK7 in oligomer disassembly, thereby enabling assembly of the canonical inflammasome disk. The authors might therefore provide mechanistic hypotheses on how NEK7 mediates dissociation and indicate which reported oligomeric forms are predicted by their landscape to be most/least susceptible to NEK7-mediated dissociation

Major comment 3 — The choice of initial inactive-state model (PDB 6NPY vs 8SXN)

The authors used PDB 6NPY as the starting structure for the inactive NLRP3 simulations rather than PDB 8SXN, justifying this choice on the grounds that 8SXN represents a dimeric, pharmacologically inhibited state complexed with MCC950. This rationale requires clarification: 6NPY itself was solved in the presence of ADP, MgCl2 and MCC950 (0.3 mM ADP, 5 mM MgCl2, 0.3 mM MCC950; Humayun Sharif et al., Nature 570:338–343, 2019). Moreover, 8SXN resolves both the N‑ and C‑lobes of NEK7 whereas 6NPY lacks part of the N‑lobe. Because these differences (such as oligomeric context and resolved regions) may influence MD and NEK7 interactions, the authors should provide a more plausible justification for choosing 6NPY as the inactive-state model. Or additional simulations (e.g., using 8SXN-derived starting structures or testing the presence/absence of MCC950) to demonstrate that the main conclusions are not dependent on the choice of initial PDB.

Correction may be needed:

NLRP3 does not contain the “N-terminal CARD domain” (Introduction)

Minor specific typos found, for example:

"NKE7" → "NEK7" (Conclusions).

"the diddle stage" → "the middle stage" (Conclusions).

"an high" → "a high" (multiple places).

"outpout density" → "output density" (Methods 5.6).

Reviewer #2: This interesting work uses molecular dynamics simulations to assess the effect of kinase NEK7 binding to NLRP3 protein on NLRP3’s ability to adopt an active state conformation. The authors find that NEK7 binding biases the NLRP3 towards more active-like states initially, but that a significant barrier remains regarding assumption of the completely formed active state. This finding is consistent with previous simulation studies, in particular that of Xu et al. (ref 16) but also provides new insight into possible intermediate states. The methods employed appear soundly executed and simulations suitably analyzed and interpreted. The results should be of interest to PLoS Comp Biol readers and I recommend the work for publication after consideration of the following remarks:

The study makes a couple of significant assumptions. The first is the study of solely monomer, as NLRP3 is generally thought to exist as oligomer in inactive and active forms. Cooperative action of the aggregate is likely to play a significant role in activation. However, interesting mechanical insights can still be derived by a reductionist study here of the monomer. In the Introduction, the discussion of experimental evidence for the (i) existence and importance of monomer and (ii) the latest thinking regarding the role of NEK7 needs to be expanded with more references.

The second assumption is the omission of cofactor for the transition simulations (but not the end point simulations). The state of the cofactor is known to be very important in activation of NLRP3. The authors also argue that omission of cofactor from both NEK7-bound and unbound forms mitigates the assumption of no cofactor, which does not seem a strong assumption. The authors also attempt to explore for a couple of snapshots the effect of this on RMSD and RMSF (quite low sensitivity measures). The two snapshots appear to have the domains at different degrees of rotation of LRR with respect to NACHT domain and thus provides something of a test (what are the angle values and time points of the samples?). What is the effect of this omission on the cofactor binding pocket residues and the adjacent domains which are very important to the hinge transition?

On a related note, it is not entirely clear why at the molecular level the active conformation has lower RMSFs and is more restricted (“intrinsically stable”) than the inactive form. The authors call this observation “remarkable”. Is this due to the presence of ATP to some degree? Some discussion of this would be useful.

Other:

Why is 6NPY far from the sampled region in Fig. 5C and 5D? Were gradually reducing constraints used to carefully equilibrate the protein? A brief comment on the resolution of the cryo-EM structures may also be useful, given their resolution is not that high.

Fig. 3(B) – why is the x-axis showing 3 us and yet 3 replicate simulations appear to be shown? Was it 3x 3 us or 3x 1 us MD simulations?

Section 2.3 - How is “loop” formation defined?

In places the language appears unduly emphatic, eg. “hallmark”, “uniquely” and “reveal”. These are predictions from MD simulations using models. Similarly the word “drive” is used in several places although this is conjecture rather than proven in the work. We note that while there clearly is electrostatic complementarity between NEK7 and NLRP3, the MMGBSA electrostatic terms of NEK7-NLRP3 largely cancel in Fig 6A; the van der Waals term is negative (the opposing nonpolar solvation term is not stated).

“diddle” should be “middle”

NKE not NEK in several places

Fig. 4 zoomed-in box is not obvious

“LINCS” not “LINC”

Cite Lopez-Blanco et al. correctly from text

Covariance equation symbols missing

**********

**Have the authors made all data and (if applicable) computational code underlying the findings in their manuscript fully available?**

The PLOS Data policy requires authors to make all data and code underlying the findings described in their manuscript fully available without restriction, with rare exception (please refer to the Data Availability Statement in the manuscript PDF file). The data and code should be provided as part of the manuscript or its supporting information, or deposited to a public repository. For example, in addition to summary statistics, the data points behind means, medians and variance measures should be available. If there are restrictions on publicly sharing data or code —e.g. participant privacy or use of data from a third party—those must be specified.

Reviewer #1: Yes

Reviewer #2: Yes

**********

PLOS authors have the option to publish the peer review history of their article (what does this mean?). If published, this will include your full peer review and any attached files.

If you choose “no”, your identity will remain anonymous but your review may still be made public.

**Do you want your identity to be public for this peer review?**     For information about this choice, including consent withdrawal, please see our Privacy Policy.

Reviewer #1: **Yes:**    Xiaodi Yu

Reviewer #2: No

**Figure resubmission:**

While revising your submission, we strongly recommend that you use PLOS’s NAAS tool (https://ngplosjournals.pagemajik.ai/artanalysis) to test your figure files. NAAS can convert your figure files to the TIFF file type and meet basic requirements (such as print size, resolution), or provide you with a report on issues that do not meet our requirements and that NAAS cannot fix.

After uploading your figures to PLOS’s NAAS tool - https://ngplosjournals.pagemajik.ai/artanalysis, NAAS will process the files provided and display the results in the "Uploaded Files" section of the page as the processing is complete. If the uploaded figures meet our requirements (or NAAS is able to fix the files to meet our requirements), the figure will be marked as "fixed" above. If NAAS is unable to fix the files, a red "failed" label will appear above. When NAAS has confirmed that the figure files meet our requirements, please download the file via the download option, and include these NAAS processed figure files when submitting your revised manuscript.

**Reproducibility:**

To enhance the reproducibility of your results, we recommend that authors of applicable studies deposit laboratory protocols in protocols.io, where a protocol can be assigned its own identifier (DOI) such that it can be cited independently in the future. Additionally, PLOS ONE offers an option to publish peer-reviewed clinical study protocols. Read more information on sharing protocols at https://plos.org/protocols?utm_medium=editorial-email&utm_source=authorletters&utm_campaign=protocols

# Author comment: Author response to Decision Letter 1

**DOI:** 10.1371/journal.pcbi.1014405.r002

6 Apr 2026

Attachment Submitted filename: point-to-point-response.pdf

# Peer review: Decision Letter 1

**DOI:** 10.1371/journal.pcbi.1014405.r003

**Authors:** Dirk Walther

4 Jun 2026

Dear Prof. Sun,

We are pleased to inform you that your manuscript 'Stage-dependent role of NEK7 in the inactive-to-active conformational transition of NLRP3 monomer' has been provisionally accepted for publication in PLOS Computational Biology.

Before your manuscript can be formally accepted you will need to complete some formatting changes, which you will receive in a follow up email. A member of our team will be in touch with a set of requests.

Please note that your manuscript will not be scheduled for publication until you have made the required changes, so a swift response is appreciated.

IMPORTANT: The editorial review process is now complete. PLOS will only permit corrections to spelling, formatting or significant scientific errors from this point onwards. Requests for major changes, or any which affect the scientific understanding of your work, will cause delays to the publication date of your manuscript.

Should you, your institution's press office or the journal office choose to press release your paper, you will automatically be opted out of early publication. We ask that you notify us now if you or your institution is planning to press release the article. All press must be co-ordinated with PLOS.

Thank you again for supporting Open Access publishing; we are looking forward to publishing your work in PLOS Computational Biology.

Best regards,

Dirk Walther, Ph.D.

Section Editor

PLOS Computational Biology

Dirk Walther

Section Editor

PLOS Computational Biology

***********************************************************

Dear authors, both reviewers are content with your revised version. Reviewer 2 suggested to reproduce Fig 5C in Fig S5, to allow an easier and direct comparison. I trust that the production process may allow updating Fig 5S accordingly, in case you wish to follow the reviewer's suggestion. Also, any reader can look at both figures side-by-side anyway.

Best regards,

Dirk Walther

Reviewer's Responses to Questions

**Comments to the Authors:**

**Please note here if the review is uploaded as an attachment.**

Reviewer #1: I have reviewed the revised version of the manuscript and the authors’ point‑by‑point responses. The authors have appropriately addressed all of my previous comments, and the quality of the manuscript has improved accordingly.

I have no further concerns and recommend the manuscript for acceptance.

Reviewer #2: The authors have satisfactorily addressed my comments and I have only one very minor remark:

Please duplicate Fig 5C into Fig S5 for ease of comparison between surfaces with and without mutations.

**********

**Have the authors made all data and (if applicable) computational code underlying the findings in their manuscript fully available?**

The PLOS Data policy requires authors to make all data and code underlying the findings described in their manuscript fully available without restriction, with rare exception (please refer to the Data Availability Statement in the manuscript PDF file). The data and code should be provided as part of the manuscript or its supporting information, or deposited to a public repository. For example, in addition to summary statistics, the data points behind means, medians and variance measures should be available. If there are restrictions on publicly sharing data or code —e.g. participant privacy or use of data from a third party—those must be specified.

Reviewer #1: Yes

Reviewer #2: Yes

**********

PLOS authors have the option to publish the peer review history of their article (what does this mean?). If published, this will include your full peer review and any attached files.

If you choose “no”, your identity will remain anonymous but your review may still be made public.

**Do you want your identity to be public for this peer review?**     For information about this choice, including consent withdrawal, please see our Privacy Policy.

Reviewer #1: **Yes:**    Xiaodi Yu

Reviewer #2: No

# Editor report: Acceptance letter

**DOI:** 10.1371/journal.pcbi.1014405.r004

**Authors:** Dirk Walther

PCOMPBIOL-D-25-02711R1

Stage-dependent role of NEK7 in the inactive-to-active conformational transition of NLRP3 monomer

Dear Dr Sun,

I am pleased to inform you that your manuscript has been formally accepted for publication in PLOS Computational Biology. Your manuscript is now with our production department and you will be notified of the publication date in due course.

The corresponding author will soon be receiving a typeset proof for review, to ensure errors have not been introduced during production. Please review the PDF proof of your manuscript carefully, as this is the last chance to correct any errors. Please note that major changes, or those which affect the scientific understanding of the work, will likely cause delays to the publication date of your manuscript.

Soon after your final files are uploaded, unless you have opted out, the early version of your manuscript will be published online. The date of the early version will be your article's publication date. The final article will be published to the same URL, and all versions of the paper will be accessible to readers.

For Research, Software, and Methods articles, you will receive an invoice from PLOS for your publication fee after your manuscript has reached the completed accept phase. If you receive an email requesting payment before acceptance or for any other service, this may be a phishing scheme. Learn how to identify phishing emails and protect your accounts at https://explore.plos.org/phishing.

Thank you again for supporting PLOS Computational Biology and open-access publishing. We are looking forward to publishing your work!

With kind regards,

Zsofia Freund

PLOS Computational Biology | Carlyle House, Carlyle Road, Cambridge CB4 3DN | United Kingdom ploscompbiol@plos.org | Phone +44 (0) 1223-442824 | ploscompbiol.org | @PLOSCompBiol
